# Bacterial Community Dynamics in Kumamoto Oyster *Crassostrea sikamea* Hatchery During Larval Development

**DOI:** 10.3389/fmicb.2022.933941

**Published:** 2022-07-12

**Authors:** Wenfang Dai, Jing Ye, Sheng Liu, Guangqiu Chang, Hongqiang Xu, Zhihua Lin, Qinggang Xue

**Affiliations:** ^1^Ninghai Institute of Mariculture Breeding and Seed Industry, Zhejiang Wanli University, Ningbo, China; ^2^Zhejiang Key Laboratory of Aquatic Germplasm Resource, College of Biological and Environmental Sciences, Zhejiang Wanli University, Ningbo, China; ^3^National Demonstration Center for Experimental Fisheries Science Education, Shanghai Ocean University, Shanghai, China

**Keywords:** *Crassostrea sikamea*, larval development, bacterial community assembly, biotic sources, functional pathway

## Abstract

Increasing evidence indicates that microbes colonized in early life stages have a long-term effect on animal wellbeing in later life stages. Related research is still limited in aquatic animals, particularly in bivalve mollusks. In this study, we analyzed the dynamics of the bacterial composition of the pelagic larval stages (fertilized egg, trochophore, D-stage, veliger, and pediveliger) and the sessile postlarval stage (spat) of Kumamoto oyster (*Crassostrea sikamea*) and their relationships with the rearing water bacterioplankton in a hatchery by using Illumina sequencing of bacterial 16S rRNA gene. Both bacterioplankton and larval bacterial communities changed greatly over larval development, and the two communities remarkably differed (*r* = 0.956, *P* < 0.001), as highlighted by the differences in the dominant taxa and bacterial diversity. Ecological processes of larval bacterial communities were measured by abundance-unweighted and abundance-weighted standardized effect sizes of the mean nearest taxon distance (ses.MNTD). The unweighted ses.MNTD analysis revealed that the deterministic process constrained the larval bacterial assembly, whereas the weighted ses.MNTD analysis showed that larval bacterial composition was initially governed by stochasticity and then gradually by determinism in the later stages. SourceTracker analysis revealed that the larval bacteria were primarily derived from an internal source, mainly from larvae at the present stage. Additionally, the abundances of larval bacterial-mediated functional pathways that were involved in the amino acid, energy, lipid and carbohydrate metabolisms significantly altered with the larval development. These findings suggest that bacteria assemble into distinct communities in larvae and rearing water in the hatchery system, and the dynamics of bacterial community composition in larvae is likely associated with larval developmental stages.

## Introduction

The Kumamoto oyster (*Crassostrea sikamea*) is an economically important marine bivalve species that is widely distributed on the coasts of East Asia, particularly in Japan, Korea, Vietnam, and China (Peng et al., 2021). The aquaculture of Kumamoto oyster is, however, still limited partially due to the lack of efficient seed production, which is frequently impacted by disease-related massive larval mortalities in hatcheries (Hedgecock et al., 1993; Banks et al., 1994). Early development of oyster is a process that involves gradual and successive changes from pelagic larvae to sessile postlarvae, and the production of healthy seeds in hatcheries depends on the normal metamorphosis of fertilized eggs to larval (i.e., trochophore, D-stage, veliger, and pediveliger) and post-larval (spat) stages (Wallace et al., 2008). It is believed that deleterious environmental factors and lack of proper nutrition constitute the most important causes of larval mortalities (Xu et al., 2011; Wang et al., 2018; Flores-Higuera et al., 2020). However, studies continue to confirm the essential roles of commensal microbiota in the survival of the larvae of aquatic animals (Boettcher et al., 2000; Xiong et al., 2017; Tarnecki et al., 2019).

Microbiota represents a vast number and variety of microbial populations that reside in, on, or around a host, and studies have revealed a close relationship between symbiotic microbes and the physiology, growth, and disease development in their host bivalves (Asmani et al., 2016; Dai et al., 2022; Paillard et al., 2022). The community assembly of animal microbiota begins after they hatch or after birth. Microbes colonized in the host's early life stages have a long-lasting effect on the microbial compositions and overall wellbeing of the host later in life (Teo et al., 2015; Pratte et al., 2018; Wang et al., 2020). As a result, increasing research efforts are devoted to understanding the factors and processes that govern the larval microbiota of aquatic species, such as shrimp (Xiong et al., 2017) and fish (Yan et al., 2016; Yukgehnaish et al., 2020). However, the information about the composition and dynamics of microbiota in bivalve mollusks during the larval developmental stages is not available to date.

Previous studies in bivalve mollusks have shown that bacterial colonization plays an important role in organ development and mechanisms preventing adverse microbes from proliferating and causing disease, and keystone bacteria colonized in larvae can be beneficial for oysters after they reach the adulthood (Prado et al., 2010; Kapareiko et al., 2011). The administration of probiotics to larvae at the early developmental stages has been reported, for example, to improve the survival rates of adult oysters possibly by inhibiting pathogens, such as *Vibrio* spp. (Prado et al., 2010; Kapareiko et al., 2011; Kesarcodi-Watson et al., 2012). In addition, the microbiota present in the digestive tracts and on the shell surfaces of oyster larvae constantly interplay with that in the rearing water during larval development, and bacteria in the external environment can rapidly colonize and become the residents of the digestive tracts and surfaces of larvae. These microbial interplays are affected by some environmental factors, such as salinity, temperature, and nutrients, in the rearing water (King et al., 2019), but the microbial assembly mechanisms in the process remain unclear.

The deterministic process and stochastic process represent the two ecological mechanisms that shape the microbial community assembly (Stegen et al., 2012). Deterministic processes determine the presence/absence and relative abundances of species, while stochastic processes assume that species show random changes in their relative abundances and probabilistic dispersal (Chase and Myers, 2011; Stegen et al., 2013). Previous studies on aquatic animals have demonstrated the essential contributions of ecological processes in structuring the gut bacterial community (Burns et al., 2016; Zhu et al., 2016). For instance, the relative contribution of the deterministic process (i.e., host selection) enhances as shrimp matures, whereas this trend is inverted as disease occurs (Xiong et al., 2018; Dai et al., 2019). In fact, the two processes have been found to simultaneously occur during local community assemblies (Xiong et al., 2017). Nonetheless, little is known regarding how these ecological processes play a role in governing the microbiota composition of oyster larvae.

In this study, we tracked the temporal variations in the bacterial community of the complete biomass (shell and soft tissues) of the pelagic larval stages (fertilized egg, trochophore, D-stage, veliger, and pediveliger) and the sessile postlarval stage (spat) of *C. sikamea*, and its corresponding rearing water from veliger to spat stages (Supplementary Figure 2). Further, we estimated the relative importance of ecological processes in governing the assembly of bacterioplankton and larval bacterial communities, and quantified the relative contributions of biotic sources to the larval bacteria. We aimed to (i) investigate the dynamics of composition, diversity, and functional potential of bacterioplankton and larval bacterial communities with the development; (ii) explore the assembly mechanisms of bacterioplankton and larval bacterial communities; and (iii) quantify the relative contributions of external and internal sources to the larval bacteria.

## Materials and Methods

### Experimental Design and Sample Collection

The 2-year-old Kumamoto oysters (*C. sikamea*) with an average shell height of 29.88 ± 0.30 mm and a total weight of 10.39 ± 0.21 g were collected from Zhejiang Province, China, on 16 June 2020 and used as the broodstock for larval production. The broodstock oysters were spawned in a hatchery at Marine Fishery Technology Innovation Research Base of Ningbo city, Zhejiang Province. Sand-filtered natural seawater without any further treatment was used for the spawning of oysters. During the whole spawning and larval nursery process, 50% of the seawater was exchanged daily and the physicochemical conditions were maintained as follows: temperature at 27 ± 3°C, salinity of 25 ± 5 psu, and dissolved oxygen at 7–8 mg/L by constant aeration. The larval feedings were done according to the developmental stages, and the feeding quantity gradually increased with the larval development. In brief, different live microalgae were used to feed larvae: *Isochrysis galbana* twice per day for the D-stage larvae, *Chaetoceros muelleri* three times per day for the earlier veliger larvae, and a mixture of *Chaetoceros muelleri* and *Tetraselmis chui* three times per day for the larvae at post-veliger to spat stages. All the microalgae used in the spawning were cultured on site.

The larvae at six developmental stages, including fertilized eggs, trochophores, D-stage, veliger, pediveliger, and spat stages, were sampled during the period of 40 days (960 h) from the indoor hatching tanks of the same size (8 × 4 × 1.3 m) according to the scheme shown in Supplementary Figures 1, 2. Simultaneously, we collected the rearing water at the corresponding stages from veliger to spat in the hatching tanks (Supplementary Figure 2). The larval developmental stages were identified under a microscope according to the morphological characteristics as previously described (Wallace et al., 2008), while the spat stage was identified visually. The collected larval samples were first mesh-filtered and washed in sterilized seawater for 10–15 s, transferred into sterilized centrifuge tubes, and followed by centrifugation at 700 rpm for 1 min at 4°C to obtain larval precipitates. The rearing water samples were filtered through a 100-μm sterilized nylon mesh to remove large planktons and particles, followed by filtration through a 0.22-μm polycarbonate membrane to collect planktonic microbial biomass. The collected larvae and membranes were immediately frozen in liquid nitrogen and stored at −80°C before DNA extraction.

### DNA Extraction, Bacterial 16S rRNA Gene Amplification, and Sequencing

Genomic DNAs (gDNAs) were extracted using the FAST DNA Spin kit in accordance with the manufacturer's instructions (Mobio Laboratories, Carlsbad, CA, USA). The DNA concentrations were quantitated by using a NanoDrop ND-2000 spectrophotometer (NanoDrop Technologies, Wilmington, USA). The extracted gDNAs served as a template for PCR amplification by targeting the V3–V4 variable regions of the bacterial 16S rRNA gene with one pair of bacteria-specific primers, 338F (5′-ACTCCTACGGGAGGCAGCA-3′) and 806R (5′-GGACTACHVGGGTWTCTAAT-3′), with Illumina adapters and dual barcodes. PCR reactions were carried out in 50 μL volumes for each sample in triplicate under the following conditions: initial denaturation at 94°C for 5 min followed by 30 cycles of denaturation at 94°C for 30 s, annealing at 53°C for 30 s, extension at 72°C for 30 s, and then final extension at 72°C for 10 min. A PCR fragment purification kit (TaKaRa, Japan) was used to purify the triplicate PCR products for each sample. The purified amplicons were analyzed for fragment size in Agilent 2100 (Agilent, USA) and quantitated using a Quant-It PicoGreen kit (Invitrogen, Thermo Fisher Scientific, USA). Same amounts of PCR amplicons from each sample were pooled and sequenced on an Illumina MiSeq platform (Illumina, San Diego, USA).

### Sequence Data Processing

The sequencing raw data were submitted to the Dix-seq platform that integrated a variety of bioinformatics tools for further analysis (Wei et al., 2020). The forward and reverse reads of the same sequence were combined using FLASH, and the chimeric sequences were further removed by UCHIME (Edgar et al., 2011). The processed sequences were then cluster-analyzed by performing USEARCH (Edgar, 2010), with 97% similarity as the threshold for the classification of an operational taxonomic unit (OTU). The most abundant sequence of an OTU was analyzed against the MicroSEQ database using UCLUST for taxonomic information and was used for taxonomic assignation by comparison with the most similar sequence in the SILVA 123 database using PyNAST (Caporaso et al., 2010). A phylogenetic tree of 16S rRNA genes was constructed from the filtered alignment using FastTree and IQ-TREE (Price et al., 2009). Sequence annotation was done using RDP Classifier (Lan et al., 2012). Next, the sequences affiliated with eukaryotic organisms, archaea, chloroplasts, and erroneous sequences were excluded from the bacterial OTU dataset. Based on OTU clustering results, α-diversity and β-diversity were analyzed using the “vegan” package in R 3.6.3.

### Data Analysis

A Venn diagram was used to calculate the number of shared and unique bacterial OTUs among samples. One-way analysis of variance (ANOVA) based on Duncan's multiple range test was used to compare the significance of differences in the bacterial α-diversity indices (including Chao1, observed species, and Shannon indices) in the larvae and rearing water among distinct developmental stages. Independent-sample *t*-test was performed to estimate the differences in the diversity of bacterial community between larvae and rearing water at same developmental stage. Non-metric multidimensional analysis (NMDS) and analysis of similarity (ANOSIM) based on Bray-Curtis distance were carried out to assess the overall differences in the bacterial community structure between larvae and rearing water in different stages of development (Clarke, 1993). The effects of developmental stage, habitat, and their interactions on the variations in bacterial community were quantitatively estimated by conducting the permutational multivariate analysis of variance (PERMANOVA) in R with the “adonis” package (R Development Core Team, 2013). Similarity percentage (SIMPER) analysis was performed to determine which OTUs drove the differences in the larval microbiota during host development. Linear discriminant analysis effect size (LEfSe) was used to identify the discriminatory taxa that were dramatically distinct in their relative abundances at each stage (Segata et al., 2011). SourceTracker model was applied to quantify the relative contributions of different biotic sources, including larval bacteria and bacterioplankton at the previous and present stages, to the larval bacterial community in R v3.6.0 with the functions “sourcetracker” and “predict” (Knights et al., 2011).

The relative importance of deterministic and stochastic processes that governed the bacterioplankton and larval bacterial community was confirmed by computing the mean nearest taxon distance (MNTD) by the “ses.mntd” function in R V3.6.3 using “picante” package (Kembel et al., 2010; Stegen et al., 2013). The ses.MNTD (standardized effect size of the phylogenetic community structure) was computed for MNTD based on the abundance-unweighted (unweighted ses.MNTD) and abundance-weighted (weighted ses.MNTD) data as reported previously (Webb et al., 2006). The MNTD was used to assess the degree of non-random phylogenetic relatedness. The deterministic and stochastic processes were judged by ses.MNTD values, that is, ses.MNTD values <-2 indicated determinism and ses.MNTD values < +2 indicated stochasticity, as described previously (Stegen et al., 2012; Dai et al., 2017).

The functional potentials of the larval bacterial communities were inferred by the Phylogenetic Investigation of Communities by Reconstruction of Observed States (PICRUSt2) (Douglas et al., 2019). The KEGG pathway categories (level 1 to level 3) were reconstructed from the KEGG annotations using the KEGG Mapper. Finally, the abundances of the KEGG modules of the larval bacterial community were visualized in R 3.6.3 using the “pheatmap” package (R Development Core Team, 2013).

## Results

### Dynamics of Bacterial Composition of Larvae and Rearing Water

**Figure 1:**
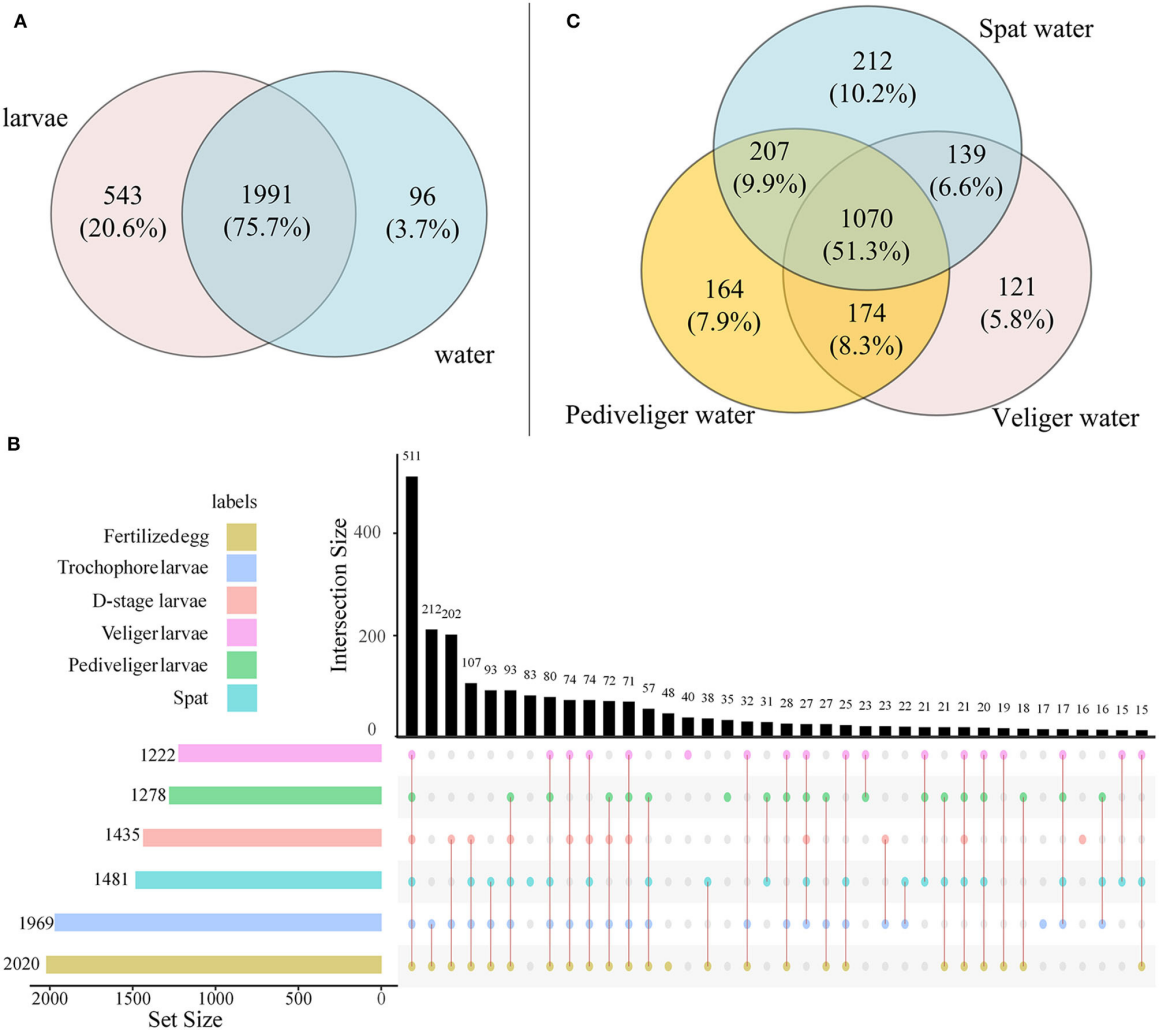
The Venn diagram shows the OTUs that are unique and shared among developmental stages in larvae and rearing water. **(A)** Number of OTUs shared between larvae and rearing water samples; **(B)** Upset plot displays the number of detected OTUs in larvae at each developmental stage (horizontal bars) and unique or shared OTUs (individual or connected points, respectively), in larvae among developmental stages; **(C)** Number of OTUs shared in rearing water among veliger, pediveliger, and spat stages.

A total of 2,045,790 high-quality reads were generated, and the mean reads of each sample were 75,770 ± 749.2 (mean ± standard deviation). After rarefaction, a total of 2,630 OTUs were identified. The shared OTUs between larvae and rearing water and among different developmental stages are depicted in Figure 1. A majority of OTUs (accounting for 75.3% of all OTUs) were shared between larvae and rearing water samples (Figure 1A). For larval bacteria, a total of 511 OTUs (20.1% of total OTUs) were shared among all six developmental stages, which accounted for 1.9, 0.6, 0.6, 1.6, 1.4, and 2.9% of fertilized egg, trochophore, D-stage, veliger, pediveliger, and spat stages, respectively (Figure 1B). For bacterioplankton, a total of 1,070 OTUs (51.3% of total OTUs) were shared among three developmental stages, which accounted for 5.7, 7.9, and 10.2% of veliger, pediveliger, and spat stages, respectively (Figure 1C).

At the bacterial (sub) phylum level, Alphaproteobacteria (38.7%), Bacteroidetes (29.3%), Gammaproteobacteria (19.8%), Firmicutes (3.8%), and Deltaproteobacteria (1.9%) were dominant in the larval bacterial community at all the six developmental stages (Supplementary Figure 3A). The bacterioplankton community at three developmental stages was predominated by Alphaproteobacteria (46.0%), Bacteroidetes (29.1%), Gammaproteobacteria (11.1%), Actinobacteria (7.0%), and Parcubacteria (3.8%) (Supplementary Figure 4A). At the finer bacterial family level, the dominant taxa were *Rhodobacteraceae* (31.4%), *Flavobacteriaceae* (27.4%), *Alteromonadaceae* (5.1%), *Moraxellaceae* (3.0%), and *Vibrionaceae* (2.4%) in the larval bacterial community (Supplementary Figure 3B), while bacterioplankton community was predominated by *Rhodobacteraceae* (38.4%), *Flavobacteriaceae* (15.3%), *Microbacteriaceae* (5.9%), *Erythrobacteraceae* (4.7%), and *Nitrosomonadaceae* (2.0%) (Supplementary Figure 4B). Statistically significant differences in the relative abundances of these dominant families were found in larvae and rearing water with larval development. For example, the relative abundance of *Alteromonadaceae* and *Vibrionaceae* decreased, while that of *Rhodobacteraceae* increased in larvae with development (Supplementary Figure 3B). Additionally, the relative abundance of *Flavobacteriaceae* first increased and afterward decreased, with the highest abundance at the D-stage (70.4%; *P* < 0.05) and lowest abundance at the spat stage (5.3%; *P* < 0.05), in the larval bacterial community over host development (Supplementary Figure 3B). However, an opposite pattern for this family was discovered in the bacterioplankton community (Supplementary Figure 4B). Notably, the relative abundance of *Rhodobacteraceae* increased in the larval bacterial community, but that of the bacterioplankton community decreased with larval development (Supplementary Figure 4B). Additionally, the relative abundances of *Microbacteriaceae* and *Erythrobacteraceae* first increased and then decreased in the bacterioplankton community over the host development (Supplementary Figure 4B). At the finer bacterial genus level, the relative abundance of *Vibrio* in the larval bacterial community was slightly higher compared to that in the corresponding bacterioplankton (Supplementary Figure 5). In particular, the relative abundance of *Vibrio* in the larval microbiota first increased with the highest abundance (5.3%; *P* < 0.05) at the trochophore stage and then decreased with the lowest abundance (0.1%; *P* < 0.05) at the veliger stage, and later was found to be stable. In contrast, *Vibrio* stabilized in the bacterioplankton community with larval development, though was found at low abundance (Supplementary Figure 5).

### Temporal Changes in Bacterial Communities of Larvae and Rearing Water

**Figure 2:**
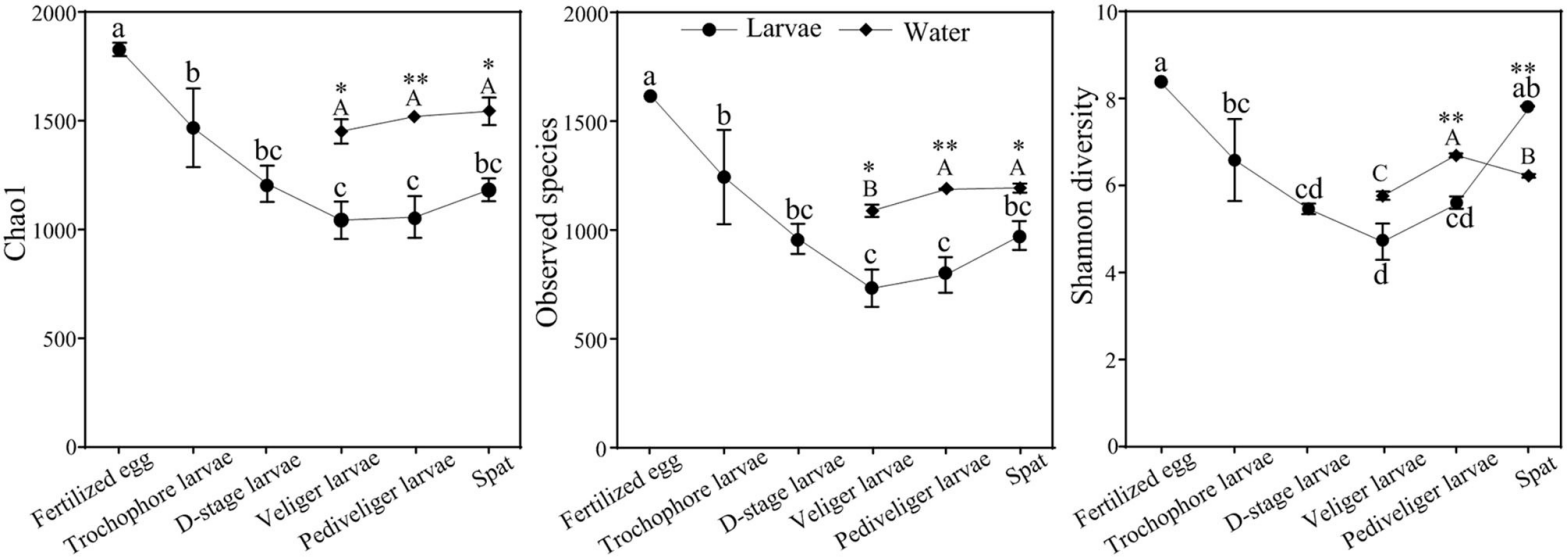
Dynamics of bacterial α-diversity of larvae and rearing water with developmental stages. Data presented represent mean ± standard error values. The different letters show significant differences among developmental stages (*P* < 0.05) based on a one-way ANOVA (lowercase for larvae and uppercase for rearing water samples). Significance of differences in the bacterial communities between larvae and rearing water was tested at same developmental stage using independent-sample *t*-test (*P < 0.05, **P < 0.01).

The bacterial α-diversity was significantly lower in larvae (*P* < 0.05) than that observed in the corresponding rearing water (Figure 2). With the development, the bacterial α-diversity of larvae strongly reduced with the lowest Chao1, observed species, and Shannon diversity indices (*P* < 0.05) at the veliger stage compared to that observed in the fertilized egg stage, and then increased. In contrast, Chao1 and observed species of bacterioplankton slightly enhanced with larval development, whereas Shannon diversity increased and then declined (Figure 2). In addition, the larval bacterial community had significantly higher (*P* < 0.05) Chao1 and observed species compared to that of the bacterioplankton community at each corresponding stage. However, an opposite trend was observed in Shannon diversity at the spat stage (Figure 2).

The NMDS ordination biplot exhibited an obvious separation of the bacterial communities between larvae and rearing water (Supplementary Figure 6). A sequential assembly of bacterial communities was observed in both larvae and rearing water over host development, as evidenced by linearly increased distances along axis 2. These findings were further corroborated by the PERMANOVA, indicating that larval developmental stage, habitat, and their interactions contributed to 48.8, 25.3, and 10.3% of variations in the bacterial communities, respectively (*P* < 0.01 in all cases) (Table 1). SIMPER indicated that the 21 top OTUs showed high contribution, which cumulatively contributed to 41.5% of the dissimilarities in larval bacterial communities among distinct developmental stages. These OTUs primarily belonged to *Rhodobacteraceae, Flavobacteriacea, Alteromonadaceae*, and *Oceanospirillaceae* taxa, of which *Rhodobacteraceae* lineage contributed to 15.0% of the total variation (Supplementary Table 1).

**Table 1:** PERMANOVA of Bray-Curits dissimilarity between samples collected at different developmental stages (fertilized egg, trochophore, D-stage, veliger, pediveliger, and spat), habitat (larvae or rearing water), and their interactions.

	**Df**	**Sum of squares**	**Mean square**	***F* model**	***R* ^2^**	***P-*Value**
Stage	5	2.76	0.55	11.23	0.488	**0.001**
Habitat	1	1.43	1.43	29.16	0.253	**0.001**
Stage × habitat	2	0.58	0.29	5.92	0.103	**0.001**
Residuals	18	0.88	0.05		0.156	
Total	26	5.65			1	

*Values in bold typeface represent significant effects at P < 0.05 level. The R^2^-values represent the proportion of the community variation constrained by each variable or their interactions*.

### Discriminatory Taxa of Larvae and Rearing Water at Distinct Developmental Stages

**Figure 3:**
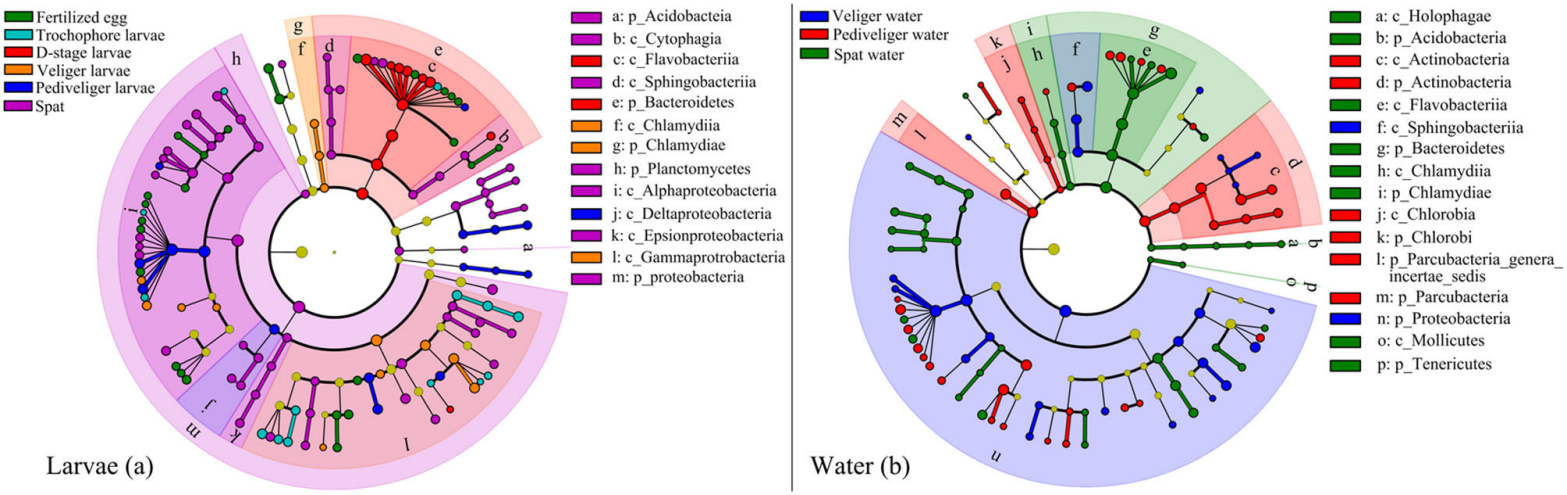
Linear discriminant analysis taxonomic cladogram shows discriminatory taxa with significant differences in their relative abundances at the OTU level in larval oyster **(a)** and rearing water **(b)** at each developmental stage. The bacterial groups affiliated from phylum to genus level are listed from center to outside. The diameter of each circle is proportional to the abundance of the bacterial taxon. Significant discriminatory nodes are colored, and branch areas are shaded according to the highest-ranked group for that taxon. If the taxon is not significantly different between the groups, the corresponding node is colored yellow. p_, phyla; c_, class.

The discriminatory taxa differed between larvae and rearing water (Figure 3). For the larval bacterial community, the discriminatory bacterial classes were Alphaproteobacteria at fertilized egg stage; Gammaproteobacteria at trochophore stage; Flavobacteriia at D-stage; Chlamydiae and Gammaproteobacteria at veliger stage; Deltaproteobacteria at pediveliger stage; and Sphingobacteriia, Alphaproteobacteria, and Epsionproteobacteria at spat stage (Figure 3a). For the bacterioplankton community, the discriminatory bacterial classes were Proteobacteria and Sphingobacteriia at the veliger stage; Chlorobia, Actinobacteria, and Parcubacteria at the pediveliger stage; and Holophagae, Flavobacteria, and Chlamydiia at the spat stage (Figure 3b). Collectively, there were most abundant bacterial discriminatory taxa in both larvae and rearing water at spat stage. In addition, 31 taxa were identified as bacterial indictors for the larval developmental stage (Supplementary Figure 7). These taxa were primarily assigned to Gammaproteobacteria, Alphaproteobacteria, and *Rhodobacteraceae*. Notably, a heatmap depicted the relative abundances of 31 indicator taxa across all samples, which showed their abilities to distinguish the samples in accordance with the developmental stage (Supplementary Figure 7).

### Ecological Processes Structuring the Bacterial Community Assembly of Larvae and Rearing Water

**Figure 4:**
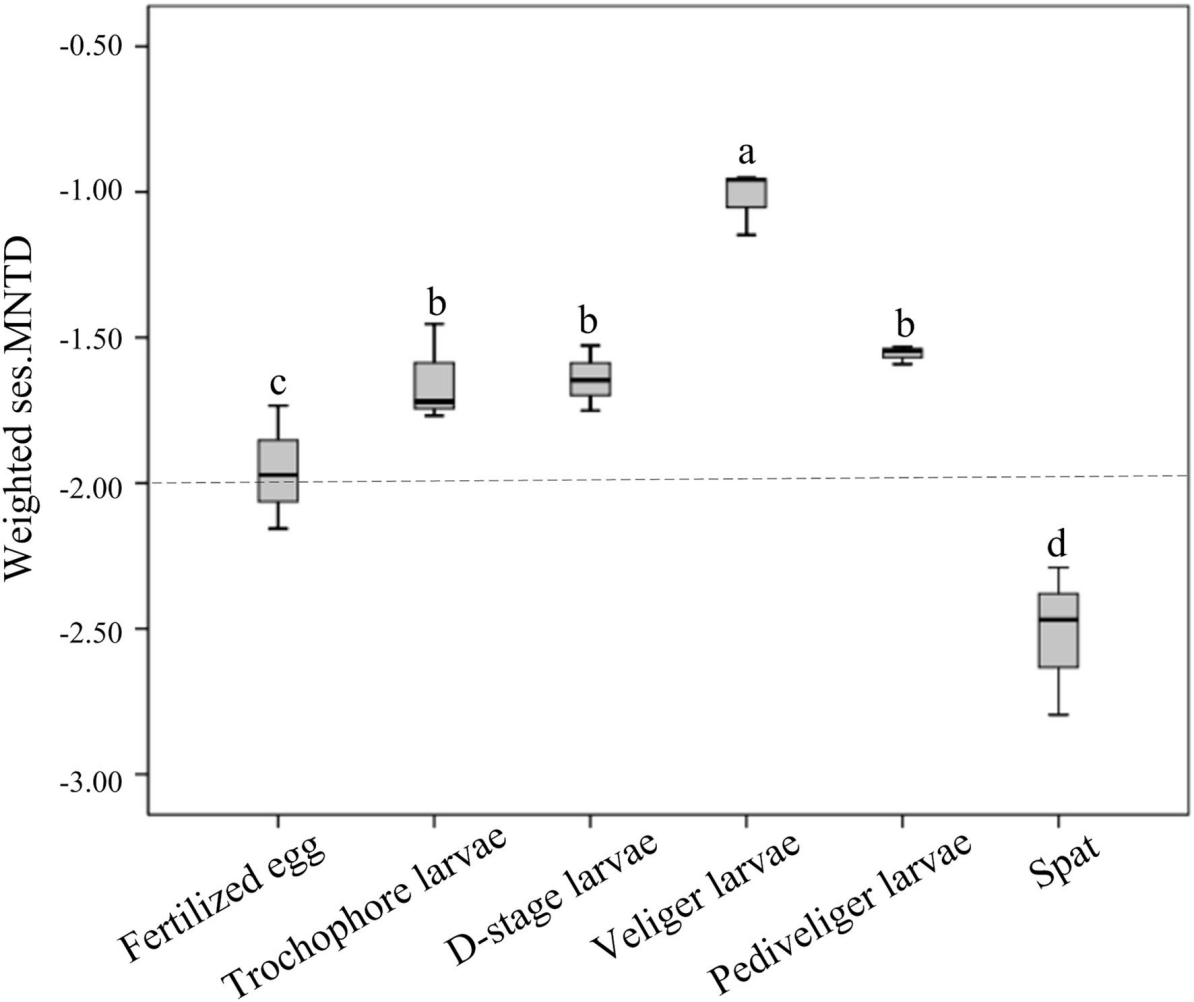
The boxplots show the ecological processes (as measured by the weighted standardized effect size of the mean nearest taxon distance, weighted ses.MNTD) of larval bacterial communities. The dotted line indicates equal roles for both. A community below the line denotes that determinism dominantly governed the bacterial community assembly, while a community above the line denotes that stochasticity was predominant. The different lowercase letters show significant differences among distinct developmental stages based on a one-way ANOVA.

The significant differences in bacterial communities between larvae and rearing water with host development (Figures 1–3; Supplementary Figures 3, 4, 6) raise the question as to which ecological processes govern this pattern. Therefore, we computed the unweighted and weighted ses.MNTD values for larval bacterial and bacterioplankton communities. Based on the abundance-unweighted data, the mean ses.MNTD values of the larval bacterial and bacterioplankton communities at all tested stages were significantly lower than zero (*P* < 0.05), and the absolute magnitude of ses.MNTD declined with larval development, suggesting that deterministic processes governed the assembly of two communities and tended to weaken with larval development (Supplementary Figures 8, 9). When taking into account information on the relative abundances of bacterial taxa, the assemblies of larval bacterial community at the fertilized egg and spat stages were primarily deterministic, as their weighted ses.MNTD values were below −2 (*t* = −16.47, *P* < 0.01; Figure 4). However, the determined assembly patterns trended to weaken from trochophore to pediveliger stages, and then the stochasticity dominated at the veliger stage (Figure 4). Thus, the relative importance of stochasticity shaping larval bacterial community decreased with advancing larval development from veliger to pediveliger stages, and the subsequent spat stage. Moreover, the assemblies of the bacterioplankton community showed an inverted V-shaped pattern over larval development, with stochasticity dominated at veliger and spat stages but determinism dominated at the pediveliger stage (Supplementary Figure 10). The absolute magnitude of ses.MNTD of the bacterioplankton community was significantly larger (*P* < 0.05) at the veliger stage than that at the spat stage, indicating that the relative influence of stochasticity in controlling the bacterioplankton community varied with larval development and was strongest at the veliger stage.

### Source of Larval Bacteria

**Figure 5:**
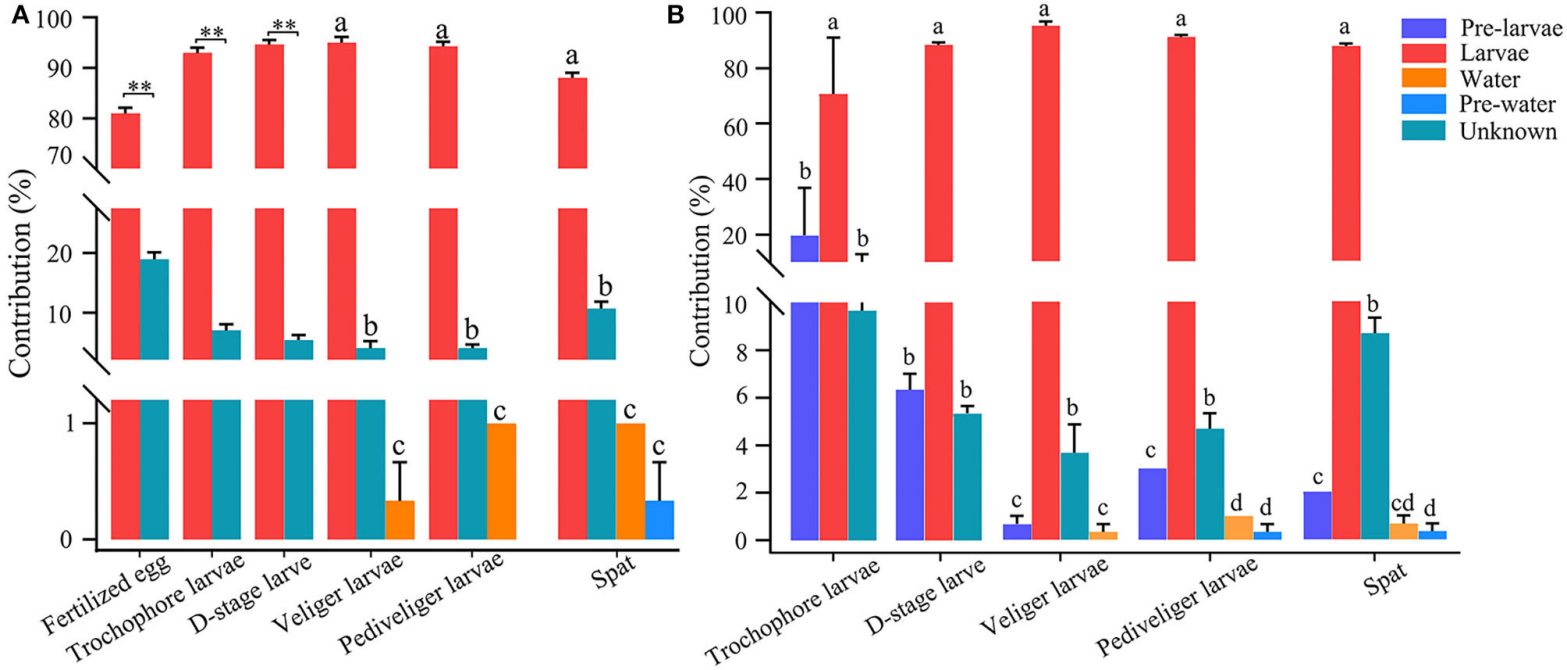
SourceTracker analysis of the relative contributions of biotic sources to larval bacteria, when not introducing **(A)** and introducing **(B)** the larval bacteria at previous stage as another of internal sources. Different lowercase letters and asterisk (**) represent significant difference (*P* < 0.05 and *P* < 0.01, respectively), among sources. Pre-larvae, larval bacteria at previous stage (i.e., larval bacteria at fertilized egg stage for trochophore larvae, larval bacteria at trochophore stage for D-stage larvae, larval bacteria at D-stage stage for veliger larvae, larval bacteria at veliger stage for prediveliger larvae, and larval bacteria at prediveliger stage for Spat); Larvae, larval bacteria at present stage; pre-water, rearing water bacterioplankton at previous stage (i.e., bacterioplankton at fertilized egg stage for trochophore larvae, bacterioplankton at trochophore stage for D-stage larvae, bacterioplankton at D-stage stage for veliger larvae, bacterioplankton at veliger stage for prediveliger larvae, and bacterioplankton at prediveliger stage for Spat); water, rearing water with bacterioplankton at present stage; unknown, unknown biotic sources.

The number of shared OTUs accounted for <50% of the total OTUs of bacterioplankton and larval bacterial communities throughout the later larval stages (Supplementary Figure 11). However, the SourceTracker analysis revealed that bacterioplankton contributed minor sources (<1%) to larval bacteria throughout the later stages (Figure 5A). Inversely, larval bacteria at the present stage contributed a major source to the composition of larval bacteria, with a relative contribution of above 80% at each developmental stage, while unknown sources contributed to <20% of the bacterial population in each developmental stage (Figure 5A). When introducing the larval bacteria at previous stage as another of internal sources, it was only second contributor to larval bacteria at the present stage, with the highest contribution of 19.8% at trochophore stage (Figure 5B). Additionally, the contribution of other sources to larval bacteria declined, such as rearing water and unknown source (Figure 5B).

### Potential Functional Profiles of Larval Bacterial Community

**Figure 6:**
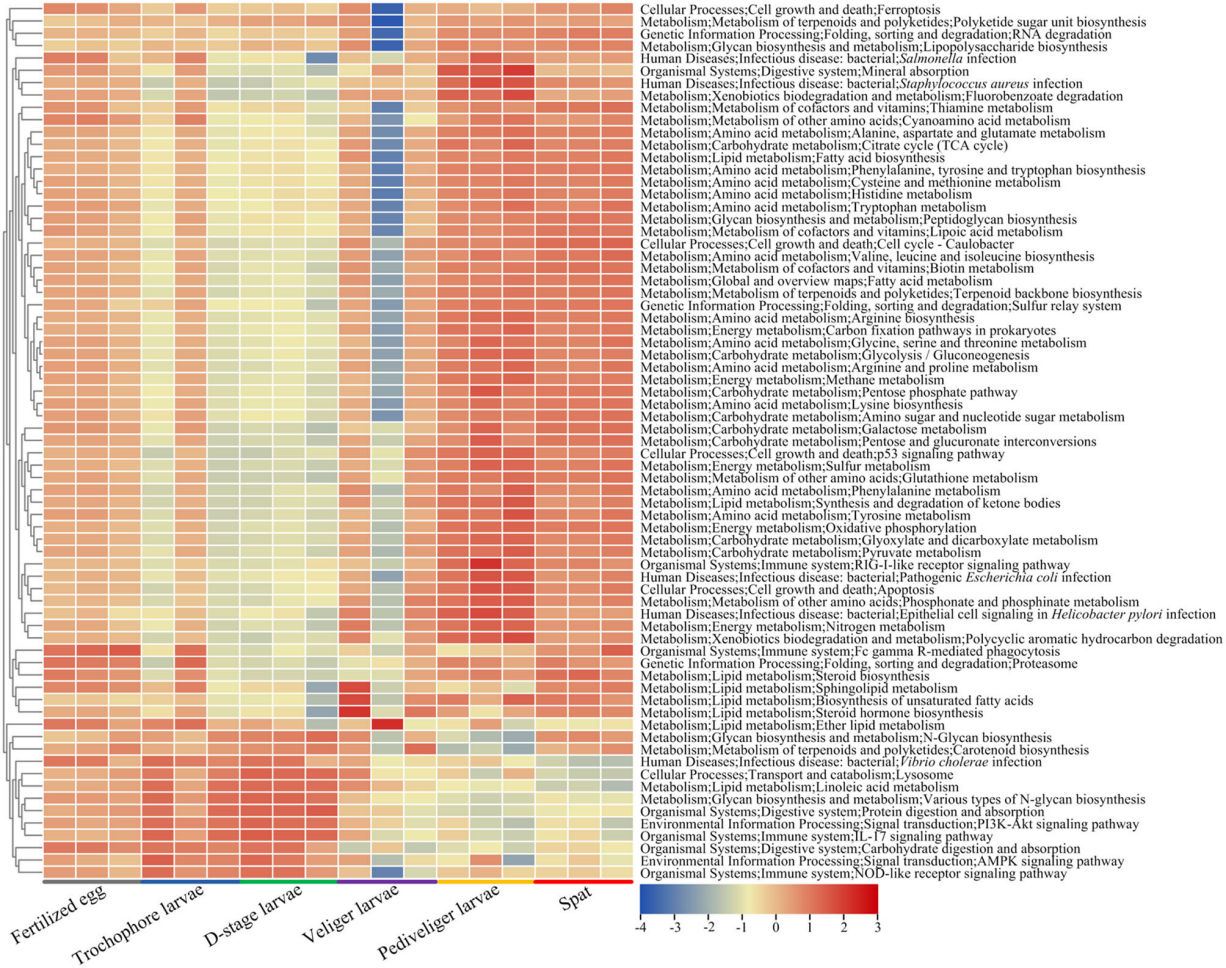
Heatmap shows the predicted functional potentials of larval bacterial community during host development. The abundances of predicted functional potentials are log_10_-transformed.

The abundances of larval microbial-mediated functional potentials exhibited clear distinction among the samples over larval development (Figure 6). The pathways involved in amino acid metabolism (alanine, aspartate and glutamate metabolism, arginine biosynthesis, cysteine and methionine metabolism, lysine biosynthesis, histidine, tryptophan, tyrosine, and phenylalanine metabolism), lipid metabolism (fatty acid and steroid biosynthesis, synthesis and degradation of ketone bodies, and sphingolipid metabolism), carbohydrate metabolism (glycolysis/gluconeogenesis, amino sugar and nucleotide sugar metabolism, galactose, pyruvate, glyoxylate, and dicarboxylate metabolism), energy metabolism (methane, sulfur and nitrogen metabolism, and oxidative phosphorylation), metabolism of terpenoids and polyketides (terpenoid backbone biosynthesis), metabolism of cofactors and vitamins (thiamine, lipoic acid, and biotin metabolism), and xenobiotic biodegradation and metabolism (fluorobenzoate degradation) first reduced with the lowest level (*P* < 0.05) at veliger stage, and then increased with the highest level (*P* < 0.05) at pediveliger stage (Figure 6). Notably, the pathways of the immune system (IL-17 and NOD-like receptor signaling pathway) and digestive system (protein and carbohydrate digestion and absorption) markedly decreased (*P* < 0.05) at the pediveliger and later stages compared to those at the D-stage, while the pathways showed an opposite trend during infections (*Staphylococcus aureus* and pathogenic *Escherichia coli* infection) (Figure 6).

## Discussion

The instability in the bacterial community of developing aquatic invertebrate larvae imparts host sensitivity to environmental disturbances, which in turn leads to larval mortality (Xiong et al., 2017; Arfken et al., 2021). Exploring the succession of larval microbiota may aid in the development of strategies for microbial management. Given the importance of early colonized microbes in the health and development of aquatic animals, a comprehensive understanding of the larval microbiota is a significant step to achieve success in oyster incubation. Currently, a few studies have assessed the effect of commensal microbes on the pelagic larval and sessile postlarval development of oysters. In this study, we explored the succession, underlying ecological mechanisms, and sources of bacterioplankton and larval bacterial communities in the Kumamoto oyster hatchery.

Bacterial α-diversity is important in promoting the stability and function of host-associated microbiota, and increased α-diversity is correlated with improved host health (McFall-Ngai et al., 2013; Clerissi et al., 2018). Therefore, bacterial diversity can serve as a biomarker of host fitness. Here, larval bacterial α-diversity of *C. sikamea* varied with host development, which was in accordance with the studies on larvae of shrimp, swimming crab, zebrafish, and southern catfish (Stephens et al., 2016; Zhang et al., 2018; Wang et al., 2020; Lu et al., 2022). We observed that larval bacterial α-diversity exhibited a U-shaped pattern along the developmental stages. Because the larvae are not fully developed, the “external” larval microbiota is primarily derived from the organs and the fraction attached to the epidermis. The “internal” gut microbiota of larvae begin to develop as the larvae start eating at the D-stage. As larval development progresses to the time of settlement and metamorphosis, modifications occur in the “internal” microbiota, primarily the gut microbiota, through reassembly of the total internal and external bacterial community. These variations could result in the dominance of gut bacteria (internal) in the larval microbiome (internal and external) and hence reduce the α-diversity of the overall larval bacterial community. Further, a low α-diversity in the host can lead to a low competition niche, which in turn implies host disease due to a less probability of having antagonistic bacteria against pathogens (Yan et al., 2012; Mallon et al., 2015). In this study, the lowest α-diversity in the larval bacterial community was observed at the veliger stage. Therefore, we inferred that the larval bacterial community was more unstable in this stage than in the other stages. Because larvae start to develop from the start of exogenous feeding at the D-stage, bacteria from the rearing water enter the larval digestive tracts, and some of them colonize its walls. Thus, the larvae at this stage could be more susceptible to environmental stress or disease. In this regard, a stable and high α-diversity is needed by the oyster larvae for disease resistance. These findings may be a key reason for the mass mortality of oyster larvae after the molting of larvae to the D-stage (Arfken et al., 2021). Additionally, a lower bacterial α-diversity was observed in the larval bacterial community compared to the bacterioplankton community during larval development in this study, indicating that host development has a greater effect on larval commensal bacteria compared with bacterioplankton. This pattern could be driven by the environmental impact on host–microbe interaction and the host-selective colonization of special functional taxa (Bakke et al., 2015; Xiong et al., 2018). Here, besides the changes in α-diversity, the bacterial community structure of *C. sikamea* larvae varied along the developmental stages, which was similar to the findings reported in shrimp, crab, and fish larvae (Stephens et al., 2016; Wang et al., 2020; Lu et al., 2022). These findings demonstrate that there is high variability in the bacterial communities of aquatic animals with host development.

Our finding that Proteobacteria was the most dominant bacterial phylum and appeared in all the development stages of *C. sikamea* larvae is in agreement with previous studies on the larval microbiota of *Crassostrea gigas* and *Crassostrea corteziensis* (Trabal Fernández et al., 2014; Asmani et al., 2016). Additionally, *Rhodobacteraceae, Flavobacteriaceae*, and *Moraxellaceae* were core taxa in the larval bacterial community, whereas this shifted to *Microbacteriaceae, Erythrobacteraceae*, and *Nitrosomonadaceae* in the bacterioplankton community. It should be noted that the colonization of alien species in the host is strictly controlled by host selection according to microbial ecology theory, thus resulting in the successful establishment of only some planktonic taxa in the host (De Schryver and Vadstein, 2014; Zhu et al., 2016; Dai et al., 2020). Consistent with the notion, we observed the difference in core microbiota between the two communities, which could be attributed to the strong selective pressure of microbial colonization in the host (Arfken et al., 2021; Lu et al., 2022). It has been proposed that the stability of core microbiota might be an important factor for host fitness, that is, dysbiosis in the microbial community generally causes host metabolic syndrome and disease (Roeselers et al., 2011; Porcellato et al., 2020). In this regard, strong selective pressure may affect the composition of host-associated microbiota, which in turn has a positive or negative impact on host fitness. The substantial changes in morphological and physiological attributes due to the metamorphic development of *C. sikamea* larvae may be associated with the variation in the bacterial community, which is attributed to the fact that commensal bacterial communities must experience reassembly parallel to biological and physiological changes, which occur during the larval and postlarval development stages. We found that *Rhodobacteraceae* maintained an overwhelming dominance within the larval bacterial community, which was inconsistent with a previous study on the postlarval microbiota of Kumamoto oyster and other oyster species (Trabal Fernández et al., 2014). *Rhodobacteraceae* population is generally distributed over various marine ecosystems. They constitute the core and beneficial bacteria that are associated with invertebrate fitness, and are a heterotrophic group of bacteria with higher diversity and multifunctionality in the degradation of organic matter, as demonstrated by their involvement in organic metabolic processes and provision of essential nutrients for host growth (Wang et al., 2020; Arfken et al., 2021). It has been demonstrated that certain *Rhodobacteraceae* lineages are beneficial for mollusk larvae and provide protection against pathogens, but other *Rhodobacteraceae* lineages cause diseases like oyster juvenile disease (Ruiz-Ponte et al., 1999; Boettcher et al., 2000; Kesarcodi-Watson et al., 2012). Inspired by these findings, we speculate that this family could play a core role and its dominance in larval microbiota is conducive to oyster development in later life. It is important to note that the relative abundance of *Vibrio* varied in larvae with host development, but *Vibrio* lineages were not abundant components of the bacterial community associated with *C. sikamea*. These findings were consistent with previous reports on the post-larvae and adults of oyster species (Trabal Fernández et al., 2014). Here, we found that the developmental stage, which reflected the phenotype of an oyster, had a significant impact on the larval bacterial community and constrained 48.8% of community variation over host development. With the alteration of oyster morphological and physiological characteristics, the larval microbiota exhibited a pattern of stage-dependent succession. Indeed, host development is one of the important factors that largely shape the larval microbiota of aquatic animals (Wang et al., 2020; Arfken et al., 2021). It is worth noting that the bacteria detected here were primarily from larval organs and epidermis. Thus, the bacterial taxa with significant variation in their abundances by LEfSe analysis likely lead to the differences in larval bacterial compositions among distinct developmental stages. Alternatively, the remarkable shift in the larval bacterial community with host development may be due to the changes in physiological status, microbial source, and feed intake pattern before and after filter feeding. This reinforces the notion that the gradual maturity of organs with the aging of the host could be more beneficial to the establishment and thriving of microbial taxa (Burns et al., 2016; Wang et al., 2020). Although larval bacterial composition varied significantly during host development, we found that more than 20% of the total OTUs in the larval bacterial community were shared between all the six developmental stages. There may exist a strong host association in the core bacterial taxa of oyster larvae. Therefore, we propose to utilize metagenomics sequencing technology for comparing the larval core taxa among developmental stages at a finer level in future work.

Previous studies have revealed the ecological processes that drive the gut microbial assembly of aquatic invertebrates (Zhu et al., 2016; Lu et al., 2022). For example, shrimp larval bacterial assembly has been shown to be controlled by environmental filtering with host development (Wang et al., 2020). Here, we quantified the relative contributions of ecological processes (determinism vs. stochasticity) in shaping the *C. sikamea* larval bacterial community assembly measured by using abundance-weighted and abundance-unweighted data. Based on the presence–absence information for bacterial taxa only, unweighted ses.MNTD showed that larval bacterial communities were governed by deterministic processes at all six developmental stages, indicating that host environmental filtering led to the assembly of bacterial communities. When taking into account their relative abundances, larval bacterial communities at the fertilized egg and spat stages were governed by deterministic process, while the stochastic process was dominant in other stages. A possible explanation for this pattern may be that the immaturity of organs in oyster larva could not prevent the colonization of external bacteria, resulting in a relatively stochastic assembly. It is worth noting that the assembly pattern dominated by the stochastic process is prone to random variation; in addition, the host is more vulnerable to harmful bacteria (Zhu et al., 2016; Xiong et al., 2017). Hence, dominant stochastic processes could result in a lower diversity and imbalance in the bacterial community during the host early life period, as observed in this study. Remarkably, the deterministic process controlling the assembly of host-associated bacterial taxa is more important in stabilizing the microbial community during the host developmental period (Burns et al., 2016). Here, larval bacteria were governed by the deterministic process at the spat stage, which might facilitate the establishment of a stable microbial community. In contrast, bacterioplankton at this stage was governed by the stochastic process, suggesting the higher dispersal ability of bacterioplankton taxa.

SourceTracker analysis enables the identification of the source of larval bacteria (Knights et al., 2011). Unexpectedly, our findings revealed that *C. sikamea* larval bacteria mainly derived from an internal source. Larval stages of *C. sikamea* are oviparous marine mollusks and are surrounded by bacterioplankton from the culture water right from their birth. Thus, it seems that culture water microbiota should contribute substantially to larval microbiota. Indeed, the larval bacterial community was markedly distinct from the bacterioplankton community and rarely sourced from rearing water, although a great number of OTUs were shared between the two communities. It could be possible that bacterial taxa with an insignificant percentage of presence can proliferate and remain as resident strains in the larval intestine, because only the bacterial groups with a minimum and significant percentage of presence are selected and those with an insignificant percentage of presence are not considered in the statistics for microbial analyses. This large difference in the bacterial communities between larvae and rearing water has also been detected in developing crab and shrimp larvae (Wang et al., 2020; Lu et al., 2022). In general, the niche preference of host-associated microorganisms does not allow sharing with bacterioplankton in a rearing environment (Walke et al., 2014; Xiong et al., 2019). Our finding may infer that *C. sikamea* larvae have a high colonization resistance against external bacteria. Following this notion, applying probiotics in culture water is likely infeasible because of the unsuccessful colonization of bacteria in oyster larvae. Thus, studies should identify probiotic bacteria from oyster larval commensal microorganisms for modulating larval growth.

The host-associated microbiota, especially gut microbiota, is often considered as an “extra organ,” due to its pivotal roles in intestinal development and physiology, as well as in host growth and health (Xiong et al., 2018; Miyamoto et al., 2019). Host energy homeostasis was maintained by utilizing nutrients and digestive activities from microbial degradation (Zhu et al., 2016; Wang et al., 2020). In this regard, the larvae with immature digestive systems partially depend on the commensal bacteria assistance for digesting food and metabolizing nutrients, which is accompanied by abundant functional potentials of multiple energy pathways involved in amino acid, lipid, carbohydrate, and energy metabolisms in larval microbiota, particularly at the fertilized egg, pediveliger, and spat stages, as predicted by PICRUSt2. However, we found that these functional pathways varied as larvae developed. This finding is partly explained by the assertion that changes in bacterial communities can translate into similar functions to maintain the necessary metabolic pathways for host growth (Zhu et al., 2016). Additionally, we found that the abundance of functional pathways involved in bacterial infectious disease increased, while that involved in digestive and immune systems decreased at the later stages of pediveliger. Under these scenarios, oyster larvae at these stages could be at a high risk of developing diseases (Arfken et al., 2021). Overall, these findings suggest that larval microbial-mediated potential functions play a significant role in the early life of oyster development.

In summary, our study demonstrated that larval development in oysters fundamentally shaped a key transition in host-associated microbial community structure, as evidenced by the temporal dynamics of larval bacterial diversity and composition. The bacterial community within larval microbiota was stochastically assembled initially but switched to deterministic assembly in the later stages of oyster development based on abundance-weighted data. The larval bacterial community was remarkably different from the bacterioplankton community, and only a small proportion of larval bacteria was sourced from their rearing water. Additionally, the functional potential of larval microbial-mediated pathways showed a high reliance on the developmental stage. This study greatly enhances our understanding of stage-specific community assembly patterns and sources in the development of *C. sikamea* larvae. Further studies are still underway to explore the possibility of improving oyster spawning practices by manipulating the microbiota in a hatchery.

## Data Availability Statement

The datasets presented in this study can be found in online repositories. The names of the repository/repositories and accession number(s) can be found at: NCBI BioProject—PRJNA847539.

## Ethics Statement

This animal study was reviewed and approved by the Institutional Animal Care and Use Committee (IACUC) of Zhejiang Wanli University, China.

## Author Contributions

QX and ZL conceived and designed the study. SL, GC, and HX collected the samples. WD and JY performed the experiments and data analysis, and wrote and revised the manuscript. All authors read and approved the manuscript.

## Funding

This study was financially supported by the National Natural Science Foundation of China (32073010), 3315 Innovative Team Project of Ningbo City, Zhejiang Province Natural Science Foundation (LQ22C190005), General Project of Zhejiang Provincial Educational Department (Y202045280), Bioengineering First-class Discipline Student Innovation Project of Zhejiang Province (CX2021049), China Agriculture Research System of MOF and MARA, and Research Plan Project of Zhejiang Wanli University.

## Conflict of Interest

The authors declare that the research was conducted in the absence of any commercial or financial relationships that could be construed as a potential conflict of interest.

## Publisher's Note

All claims expressed in this article are solely those of the authors and do not necessarily represent those of their affiliated organizations, or those of the publisher, the editors and the reviewers. Any product that may be evaluated in this article, or claim that may be made by its manufacturer, is not guaranteed or endorsed by the publisher.

## References

[B1] Arfken A. Song B. Allen S. K. Carnegie R. B. (2021). Comparing larval microbiomes of the eastern oyster (*Crassostrea virginica*) raised in different hatcheries. Aquaculture 531, 735955. 10.1016/j.aquaculture.2020.735955

[B2] Asmani K. Petton B. Le Grand J. Mounier J. Robert R. Nicolas J. L. (2016). Establishment of microbiota in larval culture of Pacific oyster, *Crassostrea gigas*. Aquaculture 464, 434–444. 10.1016/j.aquaculture.2016.07.020

[B3] Bakke I. Coward E. Andersen T. Vadstein O. (2015). Selection in the host structures the microbiota associated with developing cod larvae (*Gadus morhua*). Environ. Microbiol. 17, 3914–3924. 10.1111/1462-2920.12888 25923170

[B4] Banks M. A. McGoldrick D. J. Borgeson W. Hedgecock D. (1994). Gametic incompatibility and genetic divergence of Pacific and Kumamoto oysters, *Crassostrea gigas* and *C. sikamea*. Mar. Biol. 121, 127–135. 10.1007/BF00349481

[B5] Boettcher K. J. Barber B. J. Singer J. T. (2000). Additional evidence that juvenile oyster disease is caused by a member of the *Roseobacter* group and colonization of nonaffected animals by *Stappia stellulata*-like strains. Appl. Environ. Microb. 66, 3924–3930. 10.1128/AEM.66.9.3924-3930.2000 10966410 PMC92240

[B6] Burns A. R. Stephens W. Z. Stagaman K. Wong S. Rawls J. F. Guillemin K.. (2016). Contribution of neutral processes to the assembly of gut microbial communities in the zebrafish over host development. ISME J. 10, 655–664. 10.1038/ismej.2015.142 26296066 PMC4817674

[B7] Caporaso J. G. Bittinger K. Bushman F. D. Desantis T. Z. Andersen G. L. Knight R. (2010). PyNAST: a flexible tool for aligning sequences to a template alignment. Bioinformatics 26, 266–267. 10.1093/bioinformatics/btp636 19914921 PMC2804299

[B8] Chase J. M. Myers J. A. (2011). Disentangling the importance of ecological niches from stochastic processes across scales. Philos. T. R. Soc. B. 366, 2351–2363. 10.1098/rstb.2011.0063 21768151 PMC3130433

[B9] Clarke K. R. (1993). Non-parametric multivariate analyses of changes in community structure. Austral. Ecol. 18, 117–143. 10.1111/j.1442-9993.1993.tb00438.x

[B10] Clerissi C. de Lorgeril J. Petton B. Lucasson A. Gueguen Y. Mitta G.. (2018). Diversity and stability of microbiota are key factors associated to healthy and diseased *Crassostrea gigas* oysters. *BioRxiv. [Preprint]*. 10.1101/378125

[B11] Dai W. Dong Y. Ye J. Xue Q. Lin Z. (2022). Gut microbiome composition likely affects the growth of razor clam *Sinonovacula constricta*. Aquaculture 550, 737847. 10.1016/j.aquaculture.2021.737847

[B12] Dai W. Qiu Q. Chen J. Xiong J. (2019). Gut eukaryotic disease-discriminatory taxa are indicative of Pacific white shrimp (*Litopenaeus vannamei*) white feces syndrome. Aquaculture 506, 154–160. 10.1016/j.aquaculture.2019.03.034

[B13] Dai W. Xiong J. Zheng H. Ni S. Ye Y. Wang C. (2020). Effect of *Rhizophora apiculata* plantation for improving water quality, growth, and health of mud crab. Appl. Microbiol. Biol. 104, 6813–6824. 10.1007/s00253-020-10716-7 32514755

[B14] Dai W. Zhang J. Tu Q. Ye D. Qiu Q. Xiong J. (2017). Bacterioplankton assembly and interspecies interaction indicating increasing coastal eutrophication. Chemosphere 177, 317–325. 10.1016/j.chemosphere.2017.03.034 28319885

[B15] De Schryver P. Vadstein O. (2014). Ecological theory as a foundation to control pathogenic invasion in aquaculture. ISME J. 8, 2360–2368. 10.1038/ismej.2014.84 24892581 PMC4260705

[B16] Douglas G. M. Maffei V. J. Zaneveld J. Yurgel S. N. Brown J. R. Taylor C. M.. (2019). PICRUSt2: an improved and extensible approach for metagenome inference. BioRxiv 2019, 672295. 10.1101/672295

[B17] Edgar R. C. (2010). Search and clustering orders of magnitude faster than BLAST. Bioinformatics 26, 2460–2461. 10.1093/bioinformatics/btq461 20709691

[B18] Edgar R. C. Haas B. J. Clemente J. C. Christopher Q. Rob K. (2011). UCHIME improves sensitivity and speed of chimera detection. Bioinformatics 27, 2194–2200. 10.1093/bioinformatics/btr381 21700674 PMC3150044

[B19] Flores-Higuera F. A. Reyes-Bonilla H. Luis-Villaseñor I. E. Mazón-Suástegui J. M. Estrada-Godinez J. A. Hernandez-Cortés P.. (2020). Effect of seawater acidity on the initial development of kumamoto oyster larvae *Crassostrea sikamea* (Amemiya, 1928). J. Shellfish Res. 39, 21–30. 10.2983/035.039.0103

[B20] Hedgecock D. Banks M. A. McGoldrick D. J. (1993). The status of the Kumamoto oyster *Crassostrea sikamea* (Amemiya 1928) in US commercial brood stocks. J. Shellfish Res. 12, 215–221.

[B21] Kapareiko D. Lim H. J. Schott E. J. Hanif A. Wikfors G. H. (2011). Isolation and evaluation of new probiotic bacteria for use in shellfish hatcheries: ii. effects of a vibrio sp. probiotic candidate upon survival of oyster larvae (*Crassostrea virginica*) in pilot-scale trials. J. Shellfish Res. 30, 617–625. 10.2983/035.030.0304

[B22] Kembel S. W. Cowan P. D. Helmus M. R. Cornwell W. K. Morlon H. Ackerly D. D.. (2010). Picante: R tools for integrating phylogenies and ecology. Bioinformatics 26, 1463–1464. 10.1093/bioinformatics/btq166 20395285

[B23] Kesarcodi-Watson A. Miner P. Nicolas J. L. Robert R. (2012). Protective effect of four potential probiotics against pathogen-challenge of the larvae of three bivalves: Pacific oyster (*Crassostrea gigas*), flat oyster (*Ostrea edulis*) and scallop (*Pecten maximus*). Aquaculture 344, 29–34. 10.1016/j.aquaculture.2012.02.029

[B24] King W. L. Jenkins C. Seymour J. R. Labbate M. (2019). Oyster disease in a changing environment: decrypting the link between pathogen, microbiome and environment. Mar. Environ. Res. 143, 124–140. 10.1016/j.marenvres.2018.11.007 30482397

[B25] Knights D. Kuczynski J. Charlson E. S. Zaneveld J. Mozer M. C. Collman R. G.. (2011). Bayesian community-wide culture-independent microbial source tracking. Nat. Methods 8, 761–763. 10.1038/nmeth.1650 21765408 PMC3791591

[B26] Lan Y. Wang Q. Cole J. R. Rosen G. L. (2012). Using the RDP classifier to predict taxonomic novelty and reduce the search space for finding novel organisms. PLoS ONE 7, e32491. 10.1371/journal.pone.0032491 22403664 PMC3293824

[B27] Lu Z. Ren Z. Lin W. Shi C. Mu C. Wang C.. (2022). Succession, sources, and assembly of bacterial community in the developing crab larval microbiome. Aquaculture 548, 737600. 10.1016/j.aquaculture.2021.737600

[B28] Mallon C. A. Elsas J. D. Salles J. F. (2015). Microbial invasions: the process, patterns, and mechanisms. Trends Microbiol. 23, 719–729. 10.1016/j.tim.2015.07.013 26439296

[B29] McFall-Ngai M. Hadfield M. G. Bosch T. C. G. Carey H. V. Domazet-Lošo T. Douglas A. E.. (2013). Animals in a bacterial world, a new imperative for the life sciences. Proc. Natl. Acad. Sci. US.A. 110, 3229–3236. 10.1073/pnas.1218525110 23391737 PMC3587249

[B30] Miyamoto J. Igarashi M. Watanabe K. Karaki S. I. Mukouyama H. Kishino S.. (2019). Gut microbiota confers host resistance to obesity by metabolizing dietary polyunsaturated fatty acids. Nat. Commun. 10, 4007. 10.1038/s41467-019-11978-0 31488836 PMC6728375

[B31] Paillard C. Gueguen Y. Wegner K. M. Bass D. Pallavicini A. Vezzulli L.. (2022). Recent advances in bivalve-microbiota interactions for disease prevention in aquaculture. Curr. Opin. Biotech. 73, 225–232. 10.1016/j.copbio.2021.07.026 34571318

[B32] Peng D. Zhang S. Zhang H. Pang D. Yang Q. Jiang R.. (2021). The oyster fishery in China: trend, concerns and solutions. Mar. Policy 129, 104524. 10.1016/j.marpol.2021.104524

[B33] Porcellato D. Meisal R. Bombelli A. Narvhus J. A. (2020). A core microbiota dominates a rich microbial diversity in the bovine udder and may indicate presence of dysbiosis. Sci. Rep. 10, 21608. 10.1038/s41598-020-77054-6 33303769 PMC7729973

[B34] Prado S. Romalde J. L. Barja J. L. (2010). Review of probiotics for use in bivalve hatcheries. Vet. Microbiol. 145, 187–197. 10.1016/j.vetmic.2010.08.021 20851536

[B35] Pratte Z. A. Besson M. Hollman R. D. Stewart F. J. (2018). The gills of reef fish support a distinct microbiome influenced by host-specific factors. Appl. Environ. Microb. 84, e00063–18. 10.1128/AEM.00063-18 29453266 PMC5930318

[B36] Price M. N. Dehal P. S. Arkin A. P. (2009). FastTree: computing large minimum evolution trees with profiles instead of a distance matrix. Mol. Biol. Evol. 26, 1641–1650. 10.1093/molbev/msp077 19377059 PMC2693737

[B37] R Development Core Team (2013). R: A Language and Environment for Statistical Computing. Vienna: The R Foundation for Statistical Computing. Available online at: http://wwwR-projectorg/

[B38] Roeselers G. Mittge E. K. Stephens W. Z. Parichy D. M. Cavanaugh C. M. Guillemin K.. (2011). Evidence for a core gut microbiota in the zebrafish. ISME J. 5, 1595–1608. 10.1038/ismej.2011.38 21472014 PMC3176511

[B39] Ruiz-Ponte C. Samain J. F. Sanchez J. L. Nicolas J. L. (1999). The benefit of a *Roseobacter* species on the survival of scallop larvae. Mar. Biotechnol. 1, 52–59. 10.1007/PL00011751 10373610

[B40] Segata N. Izard J. Waldron L. Gevers D. Miropolsky L. Garrett W. S.. (2011). Metagenomic biomarker discovery and explanation. Genome Biol. 12, R60. 10.1186/gb-2011-12-6-r60 21702898 PMC3218848

[B41] Stegen J. C. Lin X. Fredrickson J. K. Chen X. Kennedy D. W. Murray C. J.. (2013). Quantifying community assembly processes and identifying features that impose them. ISME J. 7, 2069–2079. 10.1038/ismej.2013.93 23739053 PMC3806266

[B42] Stegen J. C. Lin X. Konopka A. E. Fredrickson J. K. (2012). Stochastic and deterministic assembly processes in subsurface microbial communities. ISME J. 6, 1653–1664. 10.1038/ismej.2012.22 22456445 PMC3498916

[B43] Stephens W. Z. Burns A. R. Stagaman K. Wong S. Rawls J. F. Guillemin K.. (2016). The composition of the zebrafish intestinal microbial community varies across development. ISME J. 10, 644–654. 10.1038/ismej.2015.140 26339860 PMC4817687

[B44] Tarnecki A. M. Wafapoor M. Phillips R. N. Rhody N. R. (2019). Benefits of a *Bacillus* probiotic to larval fish survival and transport stress resistance. Sci. Rep. 9, 4892. 10.1038/s41598-019-39316-w 30894554 PMC6426941

[B45] Teo S. M. Mok D. Pham K. Kusel M. Serralha M. Troy N.. (2015). The infant nasopharyngeal microbiome impacts severity of lower respiratory infection and risk of asthma development. Cell Host Microbe 17, 704–715. 10.1016/j.chom.2015.03.008 25865368 PMC4433433

[B46] Trabal Fernández N. Mazón-Suástegui J. M. Vázquez-Juárez R. Ascencio-Valle F. Romero J. (2014). Changes in the composition and diversity of the bacterial microbiota associated with oysters (*Crassostrea corteziensis, Crassostrea gigas* and *Crassostrea sikamea*) during commercial production. FEMS Microbiol. Ecol. 88, 69–83. 10.1111/1574-6941.12270 24325323

[B47] Walke J. B. Becker M. H. Loftus S. C. House L. L. Cormier G. Jensen R. V.. (2014). Amphibian skin may select for rare environmental microbes. ISME J. 8, 2207–2217. 10.1038/ismej.2014.77 24858782 PMC4992085

[B48] Wallace R. K. Waters P. Rikard F. S. (2008). Oyster Hatchery Techniques. Southern Regional Aquaculture Center, No. 4302. Auburn.

[B49] Wang T. Li Q. Zhang J. Yu R. (2018). Effects of salinity, stocking density, and algal density on growth and survival of Iwagaki oyster *Crassostrea nippona* larvae. Aquacult. Int. 26, 947–958. 10.1007/s10499-018-0261-3

[B50] Wang Y. Wang K. Huang L. Dong P. Wang S. Chen H.. (2020). Fine-scale succession patterns and assembly mechanisms of bacterial community of *Litopenaeus vannamei* larvae across the developmental cycle. Microbiome 8, 106. 10.1186/s40168-020-00879-w 32620132 PMC7334860

[B51] Webb C. O. Losos J. B. Agrawal A. A. (2006). Integrating phylogenies into community ecology. Ecology 87, S1–S2. 10.1890/0012-9658(2006)871:IPICE2.0.CO;2

[B52] Wei Y. Ren T. Zhang L. (2020). Dix-seq: an integrated pipeline for fast amplicon data analysis. BioRxiv. [Preprint]. 10.1101/2020.05.11.089748

[B53] Xiong J. Dai W. Qiu Q. Zhu J. Yang W. Li C. (2018). Response of host-bacterial colonization in shrimp to developmental stage, environment and disease. Mol. Ecol. 27, 3686–3699. 10.1111/mec.14822 30070062

[B54] Xiong J. Xuan L. Yu W. Zhu J. Qiu Q. Chen J. (2019). Spatiotemporal successions of shrimp gut microbial colonization: high consistency despite distinct species pool. Environ. Microbiol. 21, 1383–1394. 10.1111/1462-2920.14578 30828926

[B55] Xiong J. Zhu J. Dai W. Dong C. Qiu Q. Li C. (2017). Integrating gut microbiota immaturity and disease-discriminatory taxa to diagnose the initiation and severity of shrimp disease. Environ. Microbiol. 19, 1490–1501. 10.1111/1462-2920.13701 28205371

[B56] Xu F. Guo X. Li L. Zhang G. (2011). Effects of salinity on larvae of the oysters Crassostrea ariakensis, *C. sikamea* and the hybrid cross. Mar. Biol. Res. 7, 796–803. 10.1080/17451000.2011.569555

[B57] Yan Q. Li J. Yu Y. Wang J. He Z. van Nostrand J.D.. (2016). Environmental filtering decreases with fish development for the assembly of gut microbiota. Environ. Microbiol. 18, 4739–4754. 10.1111/1462-2920.13365 27130138

[B58] Yan Q. van der Gast C.J. Yu Y. (2012). Bacterial community assembly and turnover within the intestines of developing zebrafish. PLoS ONE 7, e30603. 10.1371/journal.pone.0030603 22276219 PMC3261916

[B59] Yukgehnaish K. Kumar P. Sivachandran P. Marimuthu K. Arshad A. Paray B. A.. (2020). Gut microbiota metagenomics in aquaculture: factors influencing gut microbiome and its physiological role in fish. Rev. Aquacult. 12, 1903–1927. 10.1111/raq.12416

[B60] Zhang Z. Li D. Refaey M. M. Xu W. Tang R. Li L. (2018). Host age affects the development of southern catfish gut bacterial community divergent from that in the food and rearing water. Front. Microbiol. 9, 495. 10.3389/fmicb.2018.00495 29616008 PMC5869207

[B61] Zhu J. Dai W. Qiu Q. Dong C. Zhang J. Xiong J. (2016). Contrasting ecological processes and functional compositions between intestinal bacterial community in healthy and diseased shrimp. Microb. Ecol. 72, 975–985. 10.1007/s00248-016-0831-8 27538872

