# Supplementary material for: Bacterial Community Dynamics in Kumamoto Oyster Crassostrea sikamea Hatchery During Larval Development
Source: Front Microbiol. 2022 Jul 12;13:933941. doi: 10.3389/fmicb.2022.933941 (PMC9315157; doi:10.3389/fmicb.2022.933941)

**Bacterial community dynamics in Kumamoto oyster *Crassostrea sikamea* hatchery during larval development**

Wenfang Dai^1,2,†^, Jing Ye^3,†^, Sheng Liu^1,2^, Guangqiu Chang^3^, Hongqiang Xu^1,2^, Zhihua Lin^1,2^, Qinggang Xue^1,2,*^

^1^ Ninghai Institute of Mariculture Breeding and Seed Industry, Zhejiang Wanli University, Ninghai 315604, China

^2^ Zhejiang Key Laboratory of Aquatic Germplasm Resource, College of Biological & Environmental Sciences, Zhejiang Wanli University, Ningbo 315100, China

^3^ National Demonstration Center for Experimental Fisheries Science Education, Shanghai Ocean University, Shanghai 201306, China

**Supplementary Table 1** SIMPER (similarity percentage) analysis identified the 21 top OTUs (contribution > 0.9%) that contributed the dissimilarities in larval bacterial communities among different developmental stages.

| Taxon ID | Taxonomy | Contribution (%) | Cumulative Contribution (%) | Mean abundance (%) | | | | | |
| --- | --- | --- | --- | --- | --- | --- | --- | --- | --- |
|  |  |  |  | Fertilized egg | Trochophore | D-stage | Veliger | Pediveliger | Spat |
| OTU3 | Bacteroidetes; Flavobacteriia; Flavobacteriales; *Flavobacteriaceae; Tenacibaculum* | 6.96 | 6.96 | 3.19±0.25 | 16.00±5.70 | 22.8±0.74 | 0.03±0.01 | 0.03±0.00 | 0.03±0.01 |
| OTU4 | Proteobacteria; Alphaproteobacteria; Rhodobacterales; *Rhodobacteraceae* | 4.00 | 10.95 | 0.49±0.06 | 0.56±0.06 | 0.89±0.13 | 16.8±7.29 | 4.66±1.32 | 1.45±0.12 |
| OTU5 | Proteobacteria; Alphaproteobacteria; Rhodobacterales; *Rhodobacteraceae* | 3.65 | 14.60 | 0.16±0.03 | 0.15±0.04 | 0.08±0.01 | 1.35±0.49 | 16.9±4.44 | 0.22±0.04 |
| OTU9 | Proteobacteria; Gammaproteobacteria; Pseudomonadales; *Moraxellaceae*; *Acinetobacter; Acinetobacter_baumannii* | 3.03 | 17.63 | 0.18±0.01 | 0.11±0.06 | 0.03±0.01 | 14.2±11.55 | 0.01±0.00 | 0.31±0.22 |
| OTU7 | Bacteroidetes; Flavobacteriia; Flavobacteriales; *Flavobacteriaceae*; *Tenacibaculum* | 2.63 | 20.26 | 1.31±0.13 | 5.93±2.02 | 8.74±0.57 | 0.02±0.00 | 0.02±0.00 | 0.02±0.00 |
| OTU11 | Bacteroidetes; Flavobacteriia; Flavobacteriales; *Flavobacteriaceae* | 2.01 | 22.27 | 0.06±0.02 | 0.09±0.01 | 0.10±0.01 | 7.07±3.36 | 3.68±0.93 | 0.04±0.01 |
| OTU6 | Proteobacteria; Alphaproteobacteria; Rhodobacterales; *Rhodobacteraceae*; *Donghicola; Donghicola_eburneus* | 1.90 | 24.17 | 0.65±0.05 | 0.72±0.08 | 1.32±0.25 | 1.85±0.59 | 8.03±5.19 | 0.30±0.07 |
| OTU10 | Proteobacteria; Alphaproteobacteria; Rhodobacterales; *Rhodobacteraceae* | 1.86 | 26.02 | 0.05±0.01 | 0.03±0.01 | 0.02±0.00 | 7.22±2.77 | 2.36±0.58 | 0.95±0.11 |
| OTU12 | Bacteroidetes; Flavobacteriia; Flavobacteriales; *Flavobacteriaceae*; *Flavobacterium* | 1.79 | 27.81 | 0.73±0.11 | 3.42±1.13 | 6.40±0.32 | 0.01±0.00 | 0.01±0.00 | 0.01±0.00 |
| OTU25 | Firmicutes; Bacilli; Bacillales; *Bacillales_Incertae_Sedis_XII*; *Exiguobacterium* | 1.50 | 29.31 | 6.13±0.63 | 1.78±1.42 | 0.01±0.01 | 0.01±0.00 | 0.01±0.00 | 0.01±0.01 |
| OTU8 | Proteobacteria; Alphaproteobacteria; Rhodobacterales; *Rhodobacteraceae* | 1.44 | 30.75 | 5.14±0.50 | 3.73±0.75 | 3.71±0.55 | 0.71±0.27 | 1.31±0.34 | 1.32±0.25 |
| OTU14 | Bacteroidetes; Flavobacteriia; Flavobacteriales; *Flavobacteriaceae*; *Tenacibaculum* | 1.32 | 32.07 | 0.68±0.09 | 3.11±1.00 | 4.33±0.05 | 0.02±0.00 | 0.01±0.00 | 0.02±0.00 |
| OTU13 | Proteobacteria; Gammaproteobacteria; Alteromonadales; *Alteromonadaceae*; *Marinobacter* | 1.26 | 33.33 | 0.09±0.01 | 0.19±0.04 | 0.30±0.10 | 3.06±1.37 | 3.99±0.73 | 0.07±0.02 |
| OTU20 | Proteobacteria; Gammaproteobacteria; Alteromonadales; *Alteromonadaceae*; *Alteromonas* | 1.19 | 34.52 | 1.77±0.60 | 4.55±0.91 | 0.52±0.15 | 0.03±0.01 | 0.01±0.00 | 0.05±0.00 |
| OTU15 | Proteobacteria; Alphaproteobacteria; Rhodobacterales; *Rhodobacteraceae* | 1.14 | 35.66 | 0.05±0.01 | 0.03±0.01 | 0.02±0.00 | 1.61±0.24 | 4.53±1.14 | 0.07±0.01 |
| OTU26 | Firmicutes; Bacilli; Bacillales; *Bacillaceae*; *Anoxybacillus* | 1.11 | 36.77 | 0.02±0.01 | 0.01±0.01 | 0.00±0.00 | 5.30±4.32 | 0.01±0.00 | 0.03±0.01 |
| OTU16 | Proteobacteria; Alphaproteobacteria; Rhodobacterales; *Rhodobacteraceae*; *Ruegeria* | 1.01 | 37.78 | 0.71±0.06 | 0.03±0.09 | 0.26±0.03 | 0.26±0.06 | 4.73±0.66 | 0.48±0.09 |
| OTU24 | Proteobacteria; Deltaproteobacteria | 0.98 | 38.76 | 0.03±0.01 | 0.02±0.01 | 0.01±0.00 | 1.44±0.78 | 3.75±1.35 | 0.04±0.01 |
| OTU18 | Bacteroidetes; Flavobacteriia; Flavobacteriales; *Flavobacteriaceae*; *Nonlabens* | 0.97 | 39.73 | 0.63±0.11 | 2.01±0.11 | 3.35±0.21 | 0.02±0.01 | 0.03±0.02 | 0.05±0.01 |
| OTU21 | Proteobacteria; Gammaproteobacteria; Oceanospirillales; *Oceanospirillaceae*; *Neptunomonas*; *Neptunomonas_concharum* | 0.91 | 40.63 | 0.09±0.01 | 0.06±0.02 | 0.02±0.00 | 1.65±0.45 | 3.35±0.87 | 0.17±0.04 |
| OTU23 | Bacteroidetes; Flavobacteriia; Flavobacteriales; *Flavobacteriaceae*; *Mesoflavibacter* | 0.90 | 41.54 | 0.69±0.09 | 1.53±0.54 | 3.29±0.54 | 0.02±0.01 | 0.01±0.00 | 0.01±0.00 |

**Supplementary Figure 1** Morphological pictures of larvae at different developmental stages. a: fertilized egg; b: trochophore; c: D-stage; d: veliger; e: pediveliger; f: spat.


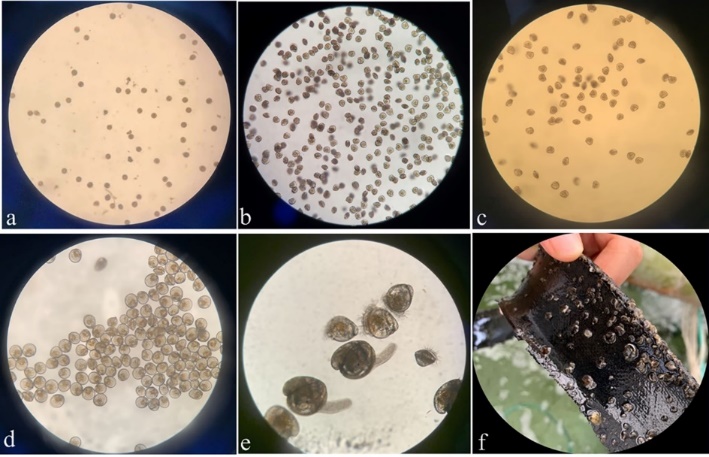


**Supplementary Figure 2** Experimental design and sampling schedule. The “Larvae” and “Water” rows showed the time point when larvae and/or rearing water samples were collected.


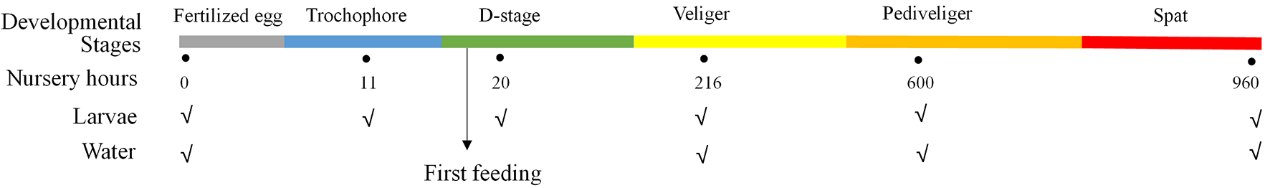


**Supplementary Figure 3** Succession of the dominant phyla (Proteobacteria is assigned to the class level) (A) and families (B) in larval bacterial community with host development. The phyla and families with mean relative abundance > 2 % in at least one group are shown.


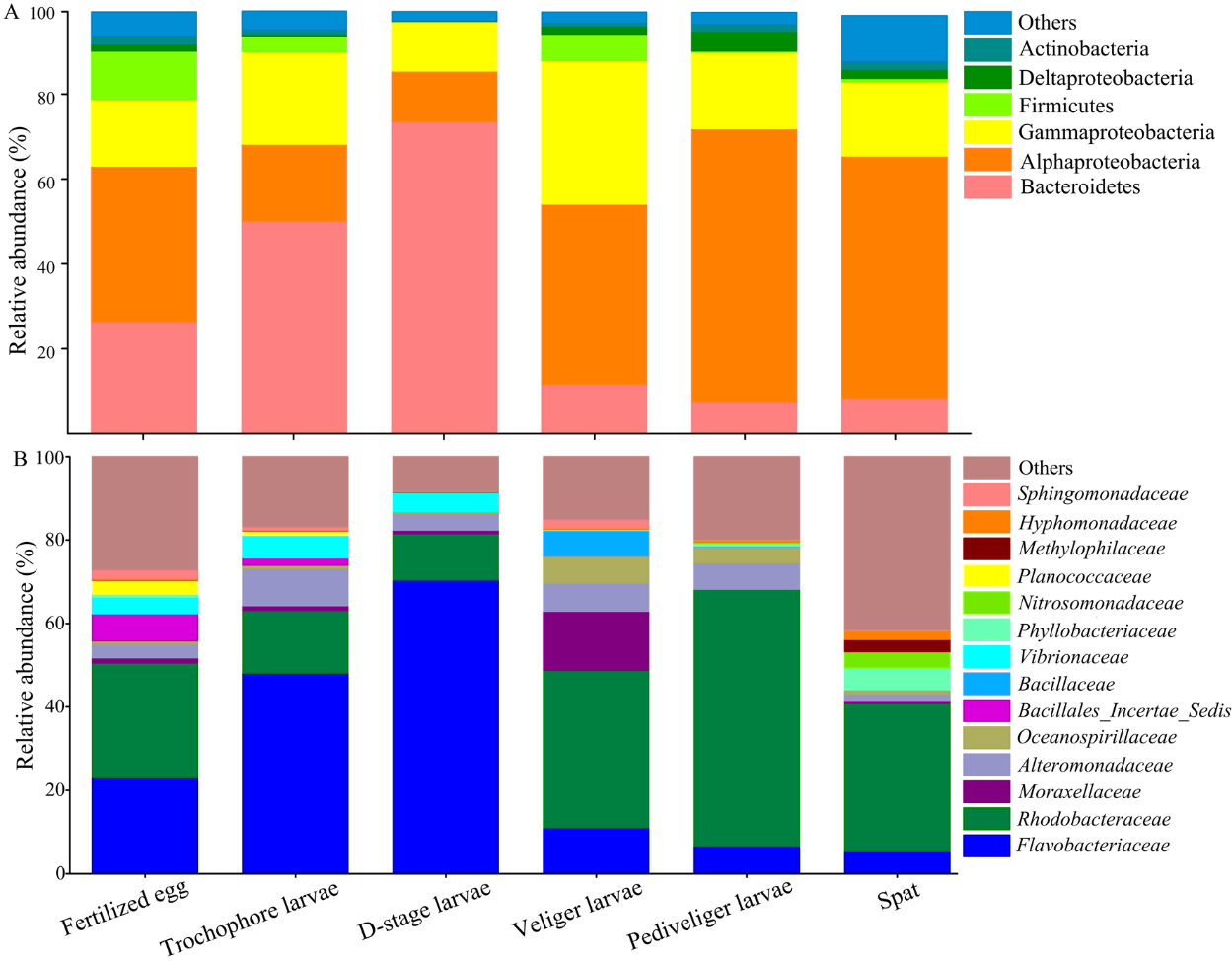


**Supplementary Figure 4** Succession of the dominant phyla (Proteobacteria is assigned to the class level) (A) and families (B) in bacterioplankton community with larval development. The phyla and families with mean relative abundance > 2 % in at least one group are shown.


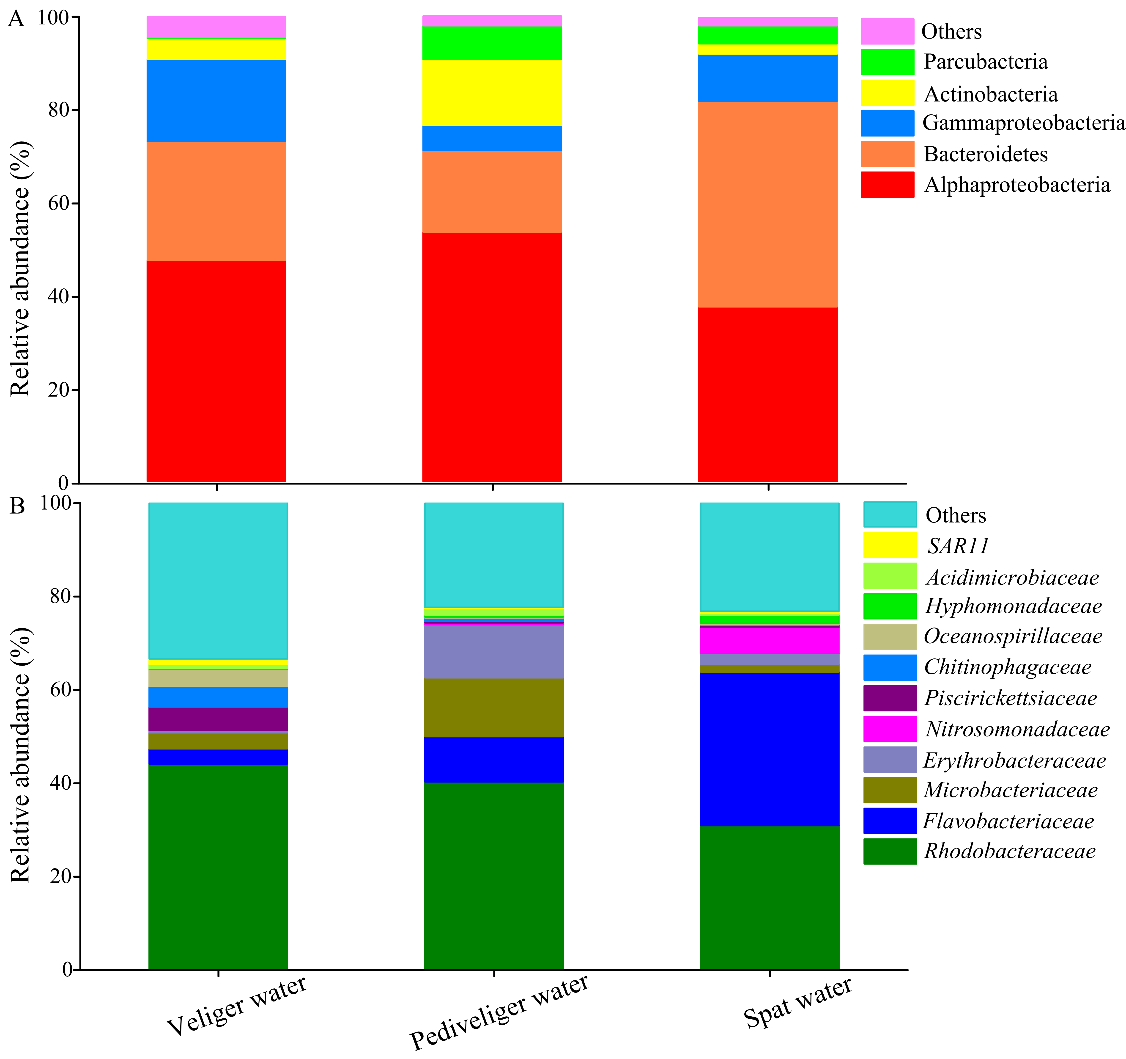


**Supplementary Figure 5** Dynamics of relative abundance of *Vibrio* in larval bacterial and bacterioplankton communities with larval development.


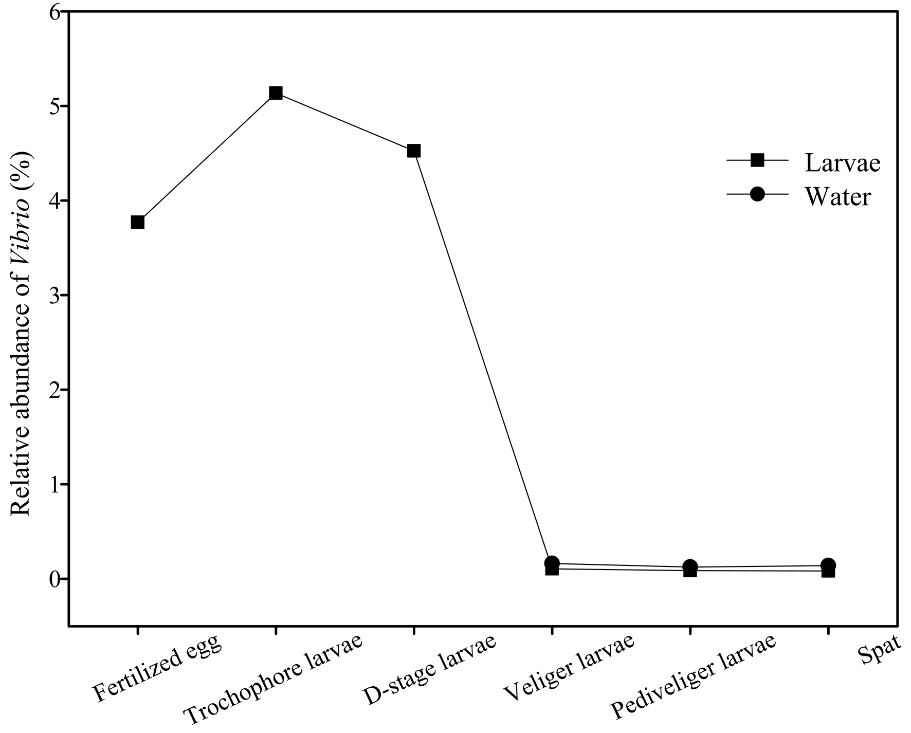


**Supplementary Figure 6** NMDS plots visualize the dissimilarities of bacterial community based on Bray-Curtis distance. The solid and open symbols indicate bacterioplankton and larval bacterial communities, respectively. Samples are coded and colored by developmental stage.





**Supplementary Figure 7** Heatmap showing the relative abundances (sqrt(x)-transformed) of 31 bacterial indicators (Indval > 0.99), with their indicator values and significance are shown in right. The branches of cluster tree are coded by the developmental stage of a corresponding sample.


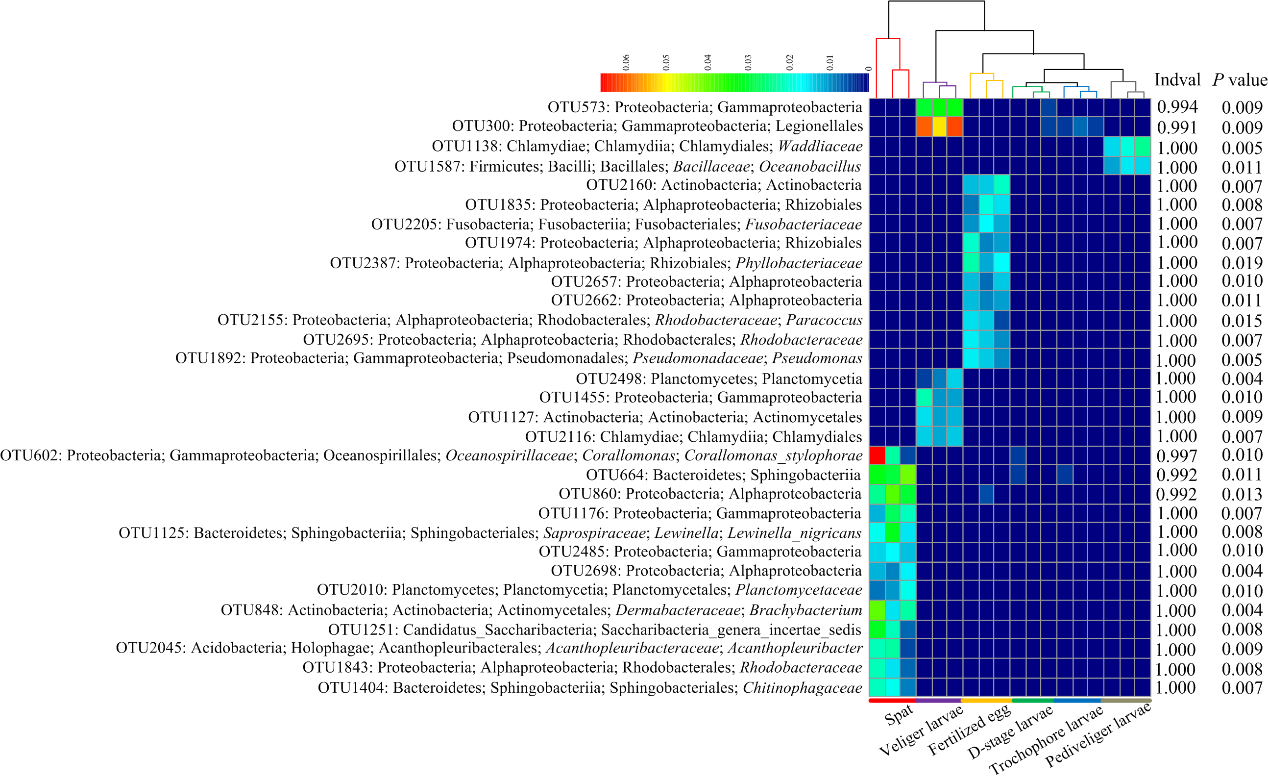


**Supplementary Figure 8** The boxplots showing the ecological processes (as measured by the unweighted standardized effect size of the mean nearest taxon distance, unweighted ses.MNTD) of larval bacterial communities along developmental stages. A community below the line indicates that deterministic process dominantly controls the community assembly, while a community above the line indicates that stochastic process is dominant. The different lowercase letters denote significant differences among distinct developmental stages based on a one-way ANOVA.


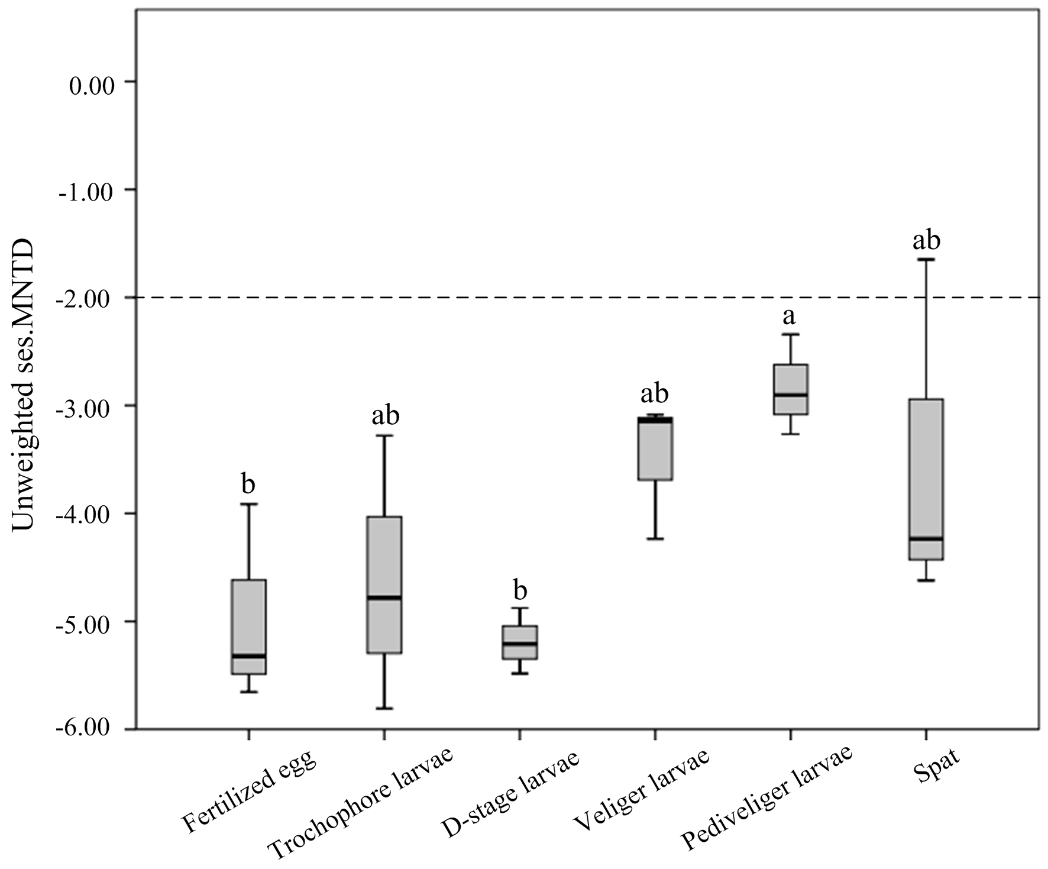


**Supplementary Figure 9** The boxplot of ecological process (as measured by unweighted ses.MNTD) of bacterioplankton communities with larval development. A community below the line indicates that deterministic process dominantly shapes the community assembly, while a community above the line indicates that stochastic process is dominant. The different lowercase letters denote significant differences among distinct developmental stages based on a one-way ANOVA.


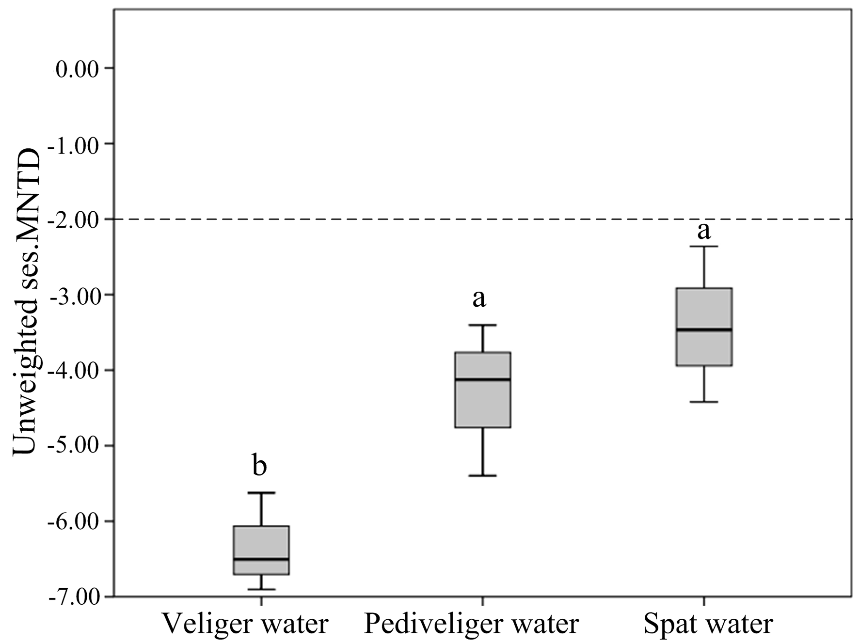


**Supplementary Figure 10** The boxplot of ecological process (as measured by weighted ses.MNTD) of bacterioplankton communities with larval development. The dashed line denotes equal roles for both. A community below the line indicates that deterministic process dominantly structures the community assembly, while a community above the line indicates that stochastic process is dominant. The different lowercase letters denote significant differences among distinct developmental stages based on a one-way ANOVA.


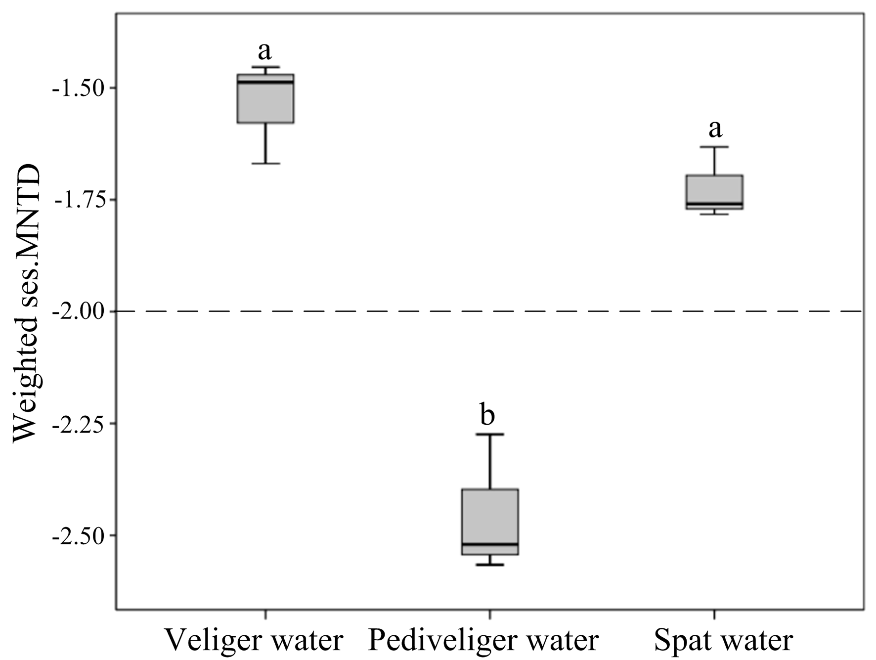


**Supplementary Figure 11** Venn diagrams showing the change in proportion of shared and unique bacterial OTUs between larvae (left) and rearing water (right) with larval development. The number in each middle dark grey circle indicates the proportion of shared bacterial OTUs between two groups; the number in each light grey circle indicates the proportion of unique bacterial OTUs in each group.


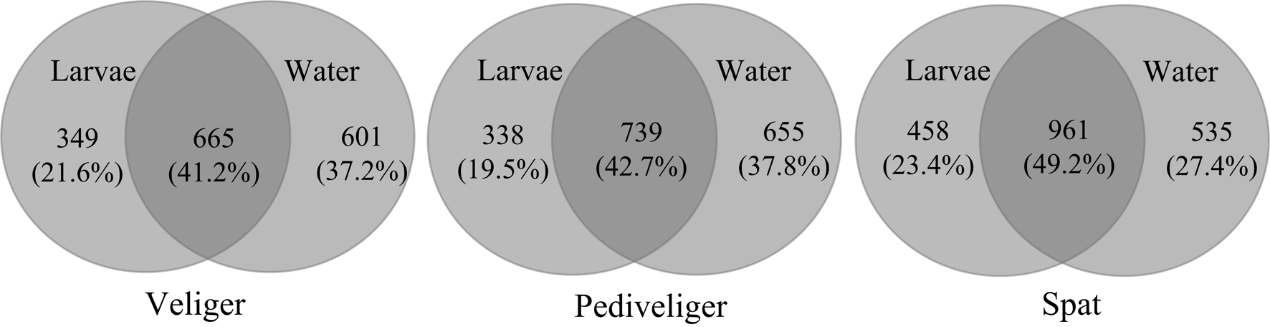

Supplement: Supplementary file 1 [file Data_Sheet_1.docx]
